# Antiphase response of the Indonesian–Australian monsoon to millennial-scale events of the last glacial period

**DOI:** 10.1038/s41598-022-21843-8

**Published:** 2022-11-23

**Authors:** Nick Scroxton, Michael K. Gagan, Linda K. Ayliffe, Wahyoe S. Hantoro, John C. Hellstrom, Hai Cheng, R. Lawrence Edwards, Jian-xin Zhao, Bambang W. Suwargadi, Hamdi Rifai

**Affiliations:** 1 Research School of Earth Sciences, The Australian National University, Canberra, ACT 2601 Australia; 2 Department of Geography, Maynooth University, Maynooth, Co. Kildare Ireland; 3 School of Earth, Atmospheric and Life Sciences, University of Wollongong, Wollongong, NSW 2522 Australia; 4 School of Earth and Environmental Sciences, The University of Queensland, St. Lucia, QLD 4072 Australia; 5 Research Center for Geotechnology, Indonesian Institute of Sciences, Bandung, 40135 Indonesia; 6 School of Earth Sciences, The University of Melbourne, Parkville, VIC 3010 Australia; 7 Institute of Global Environmental Change, Xi’an Jiatong University, Xi’an, 710049 China; 8 State Key Laboratory of Loess and Quaternary Geology, Institute of Earth Environment, Chinese Academy of Sciences, Xi’an, 710061 China; 9 Department of Earth and Environmental Sciences, University of Minnesota, Minneapolis, MN 55455 USA; 10 Department of Physics, Universitas Negeri Padang, Padang, 25131 Indonesia

**Keywords:** Climate change, Palaeoclimate

## Abstract

Antiphase behaviour of monsoon systems in alternate hemispheres is well established at yearly and orbital scales in response to alternating sensible heating of continental landmasses. At intermediate timescales without a sensible heating mechanism both in-phase and antiphase behaviours of northern and southern hemisphere monsoon systems are recorded at different places and timescales. At present, there is no continuous, high resolution, precisely dated record of millennial-scale variability of the Indonesian–Australian monsoon during the last glacial period with which to test theories of paleomonsoon behaviour. Here, we present an extension of the Liang Luar, Flores, speleothem δ^18^O record of past changes in southern hemisphere summer monsoon intensity back to 55.7 kyr BP. Negative δ^18^O excursions (stronger monsoon) occur during Heinrich events whereas positive excursions (weaker monsoon) occur during Dansgaard-Oeschger interstadials—a first order antiphase relationship with northern hemisphere summer monsoon records. An association of negative δ^18^O excursions with speleothem growth phases in Liang Luar suggests that these stronger monsoons are related to higher rainfall amounts. However, the response to millennial-scale variability is inconsistent, including a particularly weak response to Heinrich event 3. We suggest that additional drivers such as underlying orbital-scale variability and drip hydrology influence the δ^18^O response.

## Introduction

A unified hypothesis on the past response of monsoon systems to global changes in climate is being tested by new paleoclimate records on a variety of timescales. The Global-Paleo-Monsoon (GPM) concept^1^ proposes that orbital to millennial-scale variability in the strength of monsoon systems in the northern and southern hemispheres acts antiphase to each other. Most notably, this occurs at the precession scale^2–4^, despite confounding effects from changing glacial/interglacial boundary conditions^5^.

The often-suggested mechanism of a north–south translation in the mean position of the Intertropical Convergence Zone (ITCZ) via differential heating of tropical and subtropical landmasses and necessary balancing of inter-hemispheric energy flow is likely an oversimplification. Numerous dynamic and thermodynamic responses to heating^5,6^ lead to significant regional differences in monsoon rainfall patterns that may be described as offshore to onshore^7^. However, many monsoons are not associated with moisture convergence, the concept of the ITCZ as a driver of rainfall variability is less precise^8^. In these scenarios the phrase ‘tropical rainbelt’ is more appropriate.

At intermediate timescales between annual and orbital, where an antiphase sensible heating mechanism (i.e., insolation) is not obvious, the evidence for interhemispheric antiphase monsoon behaviour in the paleorecord shows considerable regional variation^5,9^, particularly outside the Atlantic sector. For example, paleoclimate records covering the last millennium from around the Indo-Pacific Warm Pool^10^ and western Indian Ocean^11^ indicate regional coherency in tropical rainfall at sites on either side of the equator at the centennial scale. Instead of meridional translation of the ITCZ, there was an expansion and contraction of the tropical rainbelt around the equator^12,13^, perhaps driven by tropical zonal variation^14^.

Paleoclimate records from the Indonesian–Australian summer monsoon (IASM) domain have potential to provide excellent tests of the GPM concept at all timescales, providing insight into the mechanisms controlling monsoon behaviour. While the South American summer monsoon and East Asian summer monsoon (EASM) show considerable interhemispheric antiphase behaviour^4^, the IASM is the direct southern hemisphere counterpart to the EASM. Potential complications to the antiphase relationship between the EASM and IASM include significant regional effects. First, changes in local sea-surface temperatures and vegetation changes may have a greater influence on IASM variability than in other monsoon systems^15^. Second, exposure of the large Sunda and Sahul shelves at times of low sea-level has an impact on the intensity of deep convection and therefore monsoon precipitation across the maritime continent^16–18^. Both Russell et al.^19^ and Krause et al.^20^ showed that glacial boundary conditions/shelf exposure played a larger role in rainfall amount variability in the heart of the Indo-Pacific Warm Pool (IPWP) than precessional forcing during the last glacial. Konecky et al.^21^ used a leaf wax dD record to suggest a decoupling of precipitation and precipitation isotopes at the precession scale in the IPWP.

At the millennial-scale, IASM paleorecords show hemispheric antiphase behaviour during the deglaciation^22^. Speleothem records from Flores, Indonesia^23^ and northern and northwestern Australia^24,25^ show negative δ^18^O excursions (increased monsoon rainfall) at the Younger Dryas (YD) and Heinrich event 1 (H1), and sediment fluxes to the Flores Sea were anomalously high during H1^26^. Overall, there is a consistent increase in monsoon strength in the IASM during the YD and H1, antiphase to the drier conditions widely recorded in EASM speleothem records from China.

The evidence for millennial-scale events in the IASM region during the last glacial is mixed. Marine sediment cores off northern Australia show periodic drying during the last glacial, potentially associated with Dansgaard-Osechger interstadials^27,28^. Terrestrial paleoclimate archives record wetter/stronger monsoon conditions for some Heinrich events^25,29–31^, and drier conditions for others^32^. So far, existing speleothem records of the IASM are discontinuous, while sediment core records frequently lack the age control to definitively assign observed variability to Heinrich events, or are influenced by climatic processes other than the IASM (e.g., ENSO)^33^. A continuous, high-resolution, precisely dated paleoclimate record of monsoon behaviour in the IASM that covers the full length of the Marine Isotope Stage (MIS) 3 millennial-scale climate variability does not currently exist. Therefore, the relationship between the IASM and EASM during millennial-scale events of the last glacial has not yet been established.

**Figure 1:**
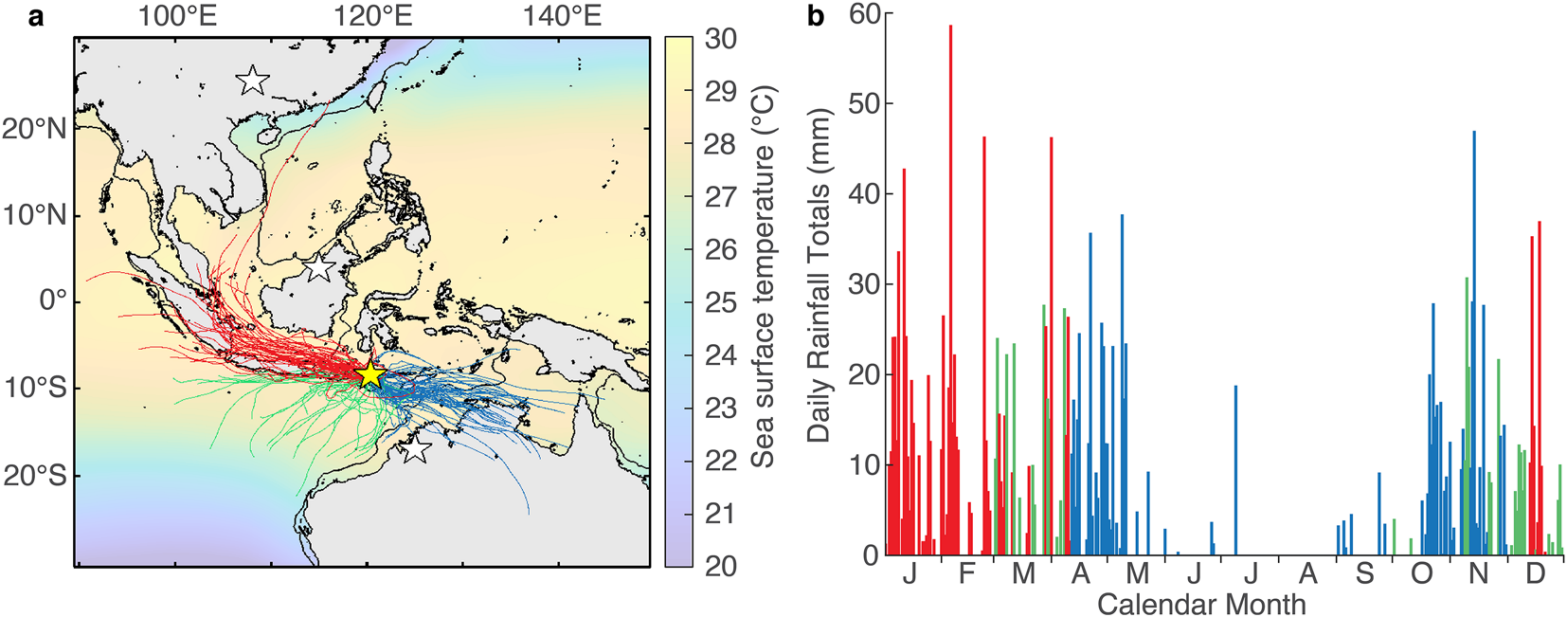
Location of Liang Luar, Flores, and the distribution of annual rainfall. (**a**) Liang Luar (yellow star) and other stalagmite δ^18^O records (white stars) discussed in the text (Dongge in China, Snail Shell Cave in Malaysian Borneo, Ball Gown Cave in Australia). Map shows mean annual sea surface temperatures (ERA-Interim 1979–2019^57^ via Ref.^58^). Black contour denotes the −120 m isobath and the approximate coastline during the Last Glacial Maximum. Coloured lines show 96-h back trajectories from 12:00 UTC on days of rainfall on Flores during 2011 using Global Assimilation System (GDAS) data and the NOAA HySplit Model^59^. Red trajectories indicate moisture from the Java Sea (IASM), blue trajectories from the Timor Sea and green trajectories from the Indian Ocean. (**b**) Daily rainfall totals for 2011, colour coded to source location as above. Rainfall data was from the NASA Tropical Rainfall Measuring Mission and extracted using the Giovanni program^60^. Map was created using MATLAB_R2019a.

In this study, we present a new continuous record of IASM variability covering the last 55.7 kyr at sub-centennial scale using speleothems from Liang Luar, Flores, Indonesia. Flores has a highly monsoonal climate defined by the seasonal movement of the tropical rainbelt. Seventy percent of annual rainfall falls during the December to March rainy season when north-westerly rains bring moisture largely sourced from the Java Sea (Fig. 1). Most of the remainder of the precipitation falls during the monsoon shoulder seasons in April, October, and November. The timing of this south-easterly derived moisture from the Savu and Timor Seas suggests this rainfall is still derived from the tropical rainbelt; from the southern limb of ITCZ convergence when the centre of convergence is to the north. The isotopic composition of this easterly rainfall is distinct^17^. The site of Liang Luar on the north side of the island, and in a north-facing valley, may bias the local rainfall in favour of the north-westerly wet season more than the 1° HySplit model would predict.

### Cave setting and speleothem samples

Liang Luar in western Flores, Indonesia (8°32’S, 120°26’E, 550 m above sea level) is a cave with a narrow entrance (~ 2 × 4 m), tight constrictions and lengthy passages (~ 1.7 km), resulting in a constant temperature (24 ± 0.5 °C) and high humidity (~ 100%), ideal for interpretation of isotopic signals in speleothems precipitated at close to isotopic equilibrium^17,23^. Good replication of δ^18^O values and trends between speleothems^23^ and a lack of positive Hendy Tests^17^ further support near-equilibrium precipitation.

**Figure 2:**
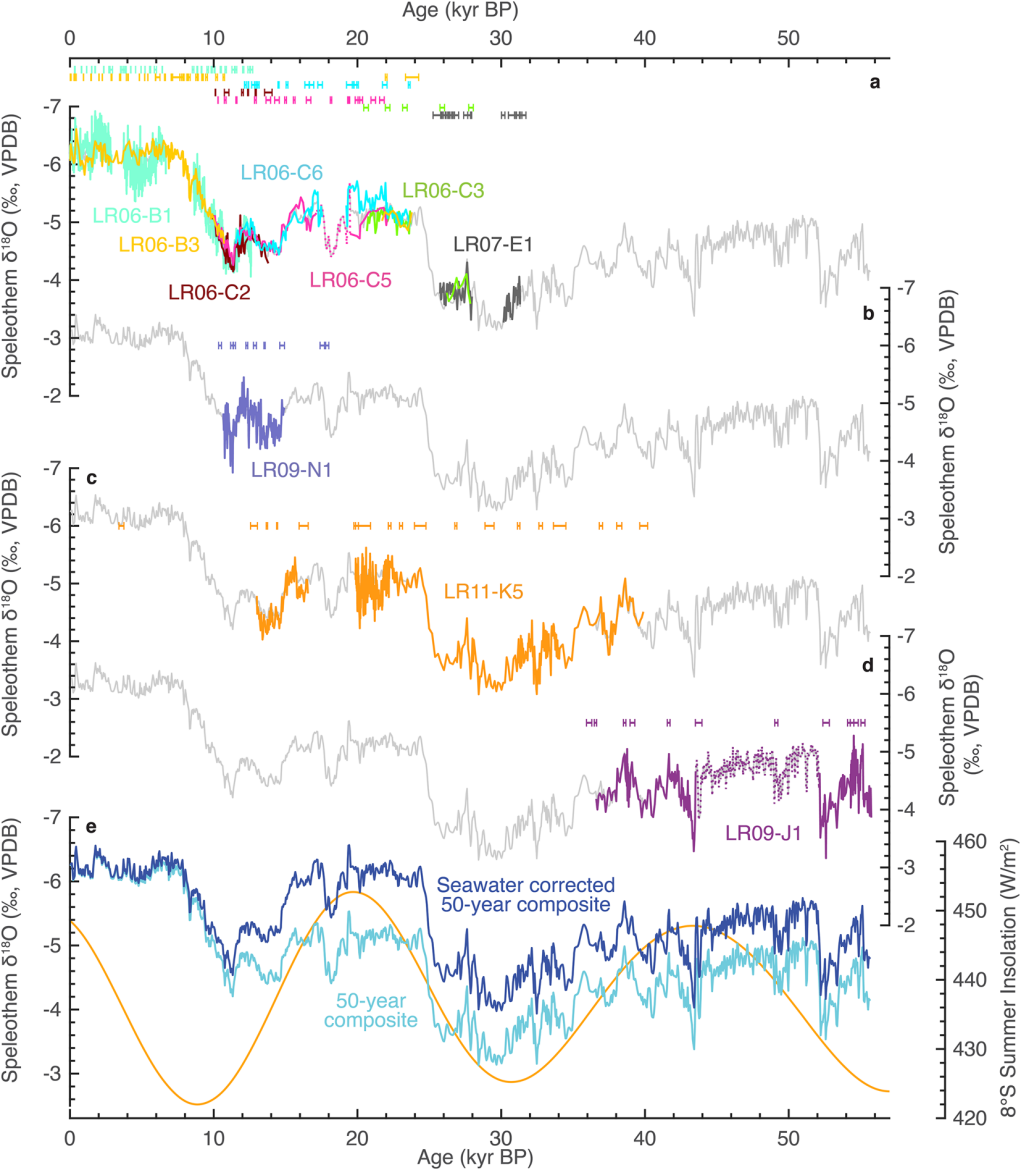
Stalagmite δ^18^O results for Liang Luar. (**a**) Previously published stalagmite δ^18^O records for Liang Luar with colour-coded 2s errors on U-Th dates^17,23,32^. New core stalagmite δ^18^O records used to construct the composite record are (**b**) LR09-N1, (**c**) LR11-K5 and (**d**) LR09-J1. Dotted lines indicate non-primary calcite mineralogies. (**e**) 50-year composite δ^18^O record and seawater corrected composite δ^18^O. Gold curve shows summer insolation at 8°S. Grey curve in (a-d) is the composite δ^18^O record.

We analysed a further eight new speleothems that build on published results for stalagmites LR06-B1, LR06-B3^17^, LR07-E1^32^, LR06-C2, LR06-C3, LR06-C5 and LR06-C6^23^. Three speleothems extend the record: stalagmites LR09-N1, LR11-K5, LR09-J1 (Fig. 2, Supplementary Figs S1–S3), and five provide valuable replication (stalagmites LR09-G4, LR09-L1, LR11-C8, LR11-G7 and flowstone LR09-E7; Supplementary Figs. S4–S8). In total there are now 34 individual growth sections from 15 speleothems from Liang Luar providing continuous coverage from 55.7 kyr BP to the present. The new speleothem records provide the length, precision, and resolution required to investigate the timing and polarity of IASM variability during the last glacial period, and therefore determine potential interhemispheric asymmetry, or lack thereof.

### Orbital-scale variability

The first-order signal of the new Liang Luar speleothem δ^18^O record is a glacial/interglacial shift to lower δ^18^O during the Holocene (Fig. 2). Removal of the seawater δ^18^O component largely negates this offset with corrected δ^18^O between 24 and 16 kyr BP comparable to the Holocene. Periods of low δ^18^O occur at 52–35, 25–15 and 9–0 kyr BP. Periods of high δ^18^O occur at 35–25 and 15–9 kyr BP. δ^18^O maxima at 30 and 10.5 kyr BP are approximately coincident with southern summer insolation lows and the δ^18^O minimum at 20 kyr BP is approximately coincident with an insolation high. These results are consistent with the phasing of speleothem δ^18^O and summer insolation in the northern hemisphere EASM^34,35^. However, the long interval of relatively low δ^18^O from 53 to 35 kyr BP is complicated by the presence of non-primary calcite, which causes an uncertain isotopic offset in the record (Supplementary Fig. S3).

**Figure 3:**
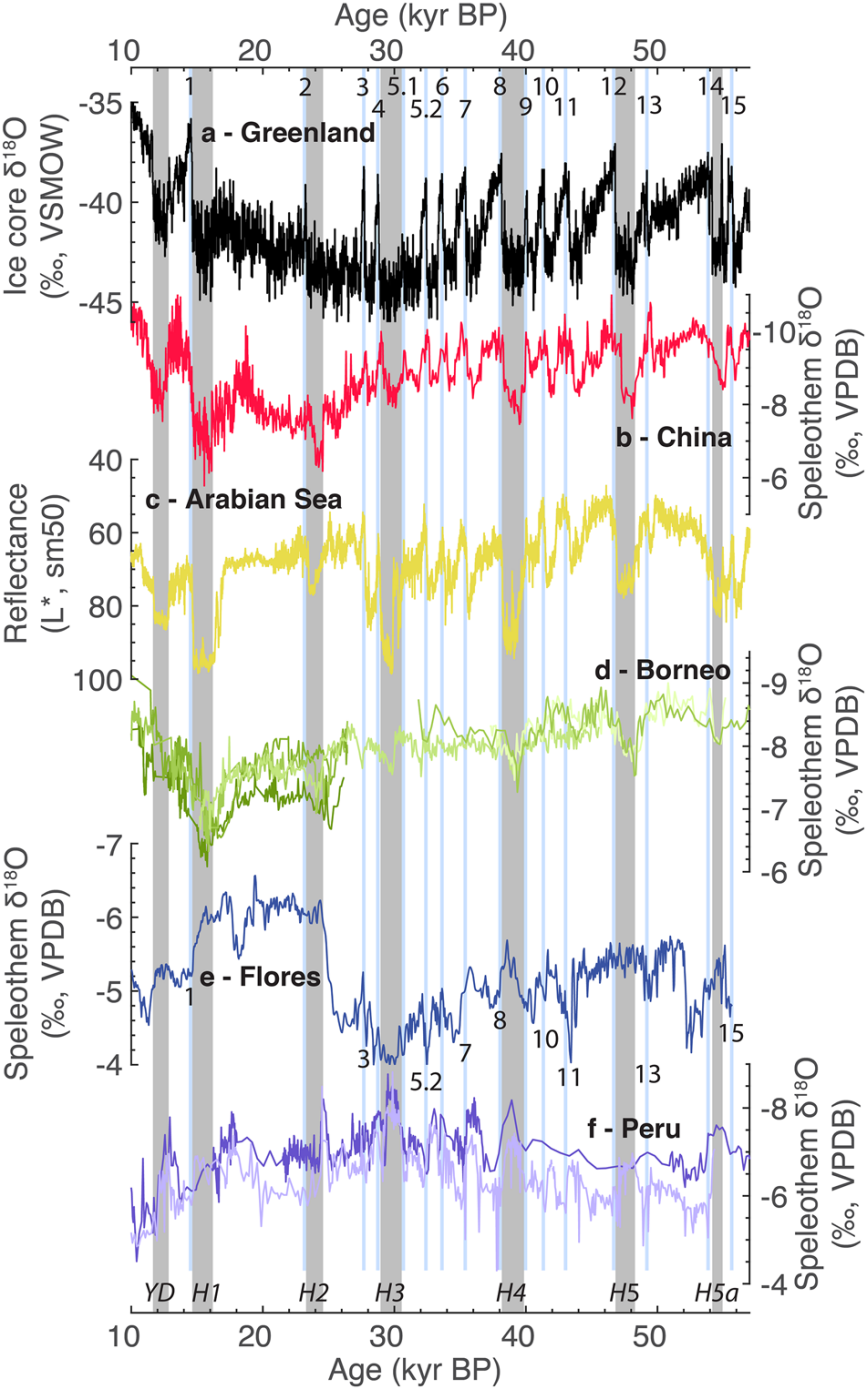
Millennial-scale events of the last glacial. (**a**) Greenland NGRIP ice core record on the GICC05/INTIMATE timescale^47^. Dansgaard-Oeschger interstadials (blue bars) are numbered. (**b**) Hulu-Sanbao-Dongge speleothem composite for China^34^. (**c**) Sediment core reflectance for the Arabian Sea^42^. (**d**) Speleothem records for Gunung Mulu National Park, Borneo^44,61^, (**e**) Liang Luar speleothem composite record, adjusted for seawater δ^18^O (this study). Possible responses to Dansgaard-Oeschger interstadials are numbered. (**f**) Speleothem records for Cueva del Diamante and El Condor Cave in Peru^40^. Grey bars indicate the five canonical Heinrich events of the last 56 kyr BP, plus the Younger Dryas and Greenland Stadial GS15.1, also known as Heinrich 5a. The timing of these events is derived from U-Th dated positive isotope excursions in the speleothem record for China.

We interpret low frequency monsoon variability as being driven by both precession and additional higher-latitude orbital, ice-sheet or shelf-exposure forcing. Between 25.4 and 18.6 kyr BP a low, and relatively steady, δ^18^O excursion around −6‰ appears unique to Flores (Fig. 3). During this period sea-level reached its lowest point and the exposure of the Sunda and Sahul shelves was at its maximum^16^. Therefore, either the continentality of rainfall at Flores would be at its greatest, potentially leading to the more negative δ^18^O state, or there was a change in the proportion of different moisture sources to those not seen in the modern. The 56–31 kyr BP precession cycle has a reduced amplitude of variability relative to the 31–9 kyr BP precession cycle. This reduced amplitude is also observed in China^34,35^ and suggests either a northern hemisphere higher latitude influence via the winter monsoon^15^, or an independent sensitivity to obliquity forcing^36^. Precise orbital, ice sheet and shelf-exposure mechanisms, seasonality influences or latitudes of forcing are unlikely to be resolved without much longer records spanning multiple glacial-interglacial and precessional cycles.

### Millennial-scale variability during the deglaciation

The response of the IASM to Heinrich 1 (H1) and the Younger Dryas (YD), as recorded by stalagmites from Liang Luar was first reported by Ayliffe et al.^23^. The addition of LR09-N1 and LR11-K5 provides excellent replication of MIS2 millennial-scale variability recorded in different areas of the cave. Our results do not significantly change the interpretation of Ayliffe et al.^23^ (Supplementary Figs. S9–S12). In short, there are significant negative δ^18^O anomalies replicated amongst multiple speleothems for both H1 and the YD that are easily distinguishable from a concurrent long-term deglaciation trend (Fig. 2). In addition, there are positive δ^18^O anomalies during the Extrapolar Climate Reversal (ECR), Bølling-Allerød (B-A) and early Holocene (eH). These changes are antiphase to those typically seen in the northern hemisphere EASM (Fig. 3).

### Heinrich events

To the first order, the Flores speleothem record shows a series of negative δ^18^O anomalies coincident with most Heinrich events of the last glacial period (Fig. 3). These excursions are recognisable in the context of other regional records. Negative δ^18^O excursions are antiphase to the Hulu-Sanbao-Dongge speleothem δ^18^O composite record for China in the northern hemisphere tropics, and in-phase with speleothem δ^18^O records for Peru in the southern hemisphere tropics. The timing of antiphase variability shows a stronger affinity with speleothem δ^18^O records for China than with NGRIP ice core δ^18^O. This could either indicate some role of the East Asian winter monsoon in IASM millennial-scale events^37,38^, or may be due to chronological uncertainty between paleoclimate records.

However, the isotopic response is not uniform among events. Heinrich events 4 and 5a show clear negative δ^18^O anomalies. Heinrich events 2 and 5 show large (~ 1‰) negative δ^18^O excursions at their onset but little change at their terminations. Heinrich event 3 shows either no signal or a positive δ^18^O signal. It should also be noted that the magnitude of Flores speleothem δ^18^O anomalies are sometimes difficult to distinguish from other (presumably non-North Atlantic forced) millennial-scale variability during MIS 2 and 3.

### Dansgaard-Oeschger events

It is also possible to recognise positive δ^18^O excursions in the Flores speleothem record coincident with Dansgaard-Oeschger (DO) interstadials (Fig. 3). Notable positive δ^18^O excursions include DO-1, 3, 5.2, 7, 8, 10, 11, 13 and 15. However, the isotopic response is inconsistent. Some DO interstadials have very muted δ^18^O responses (DO-5.1), and some produce no response (DO-2, 9, 12). Frequently, age model uncertainty does not allow us to distinguish between negative and positive δ^18^O anomalies (DO-4, 5.1, 6 and 14). Previously, a positive δ^18^O excursion during DO-21 was identified and replicated in stalagmites from Liang Luar with high resolution of age determinations^39^ (> 5 U-Th determinations per 2000 years). Therefore, while we have confidence in a positive δ^18^O response to DO events, a combination of muted response for some events and age model uncertainty prevents us from 1:1 assignment of DO interstadials to positive δ^18^O excursions in the Flores speleothem record.

### Interpretation of speleothem δ^18^O

The new record shows antiphase behaviour with EASM monsoon records in the northern hemisphere such as the Hulu-Sanbao-Dongge speleothem record for China^34,35^ and in-phase behaviour in speleothem δ^18^O records for El Condor Cave and Cueva del Diamante in Peru^40^ (Fig. 3) and Caverna Botuverá in Brazil^41^. We interpret negative δ^18^O anomalies in the Flores speleothem δ^18^O record are interpreted as increased regional monsoon convection in the IASM. The δ^18^O anomalies at Flores (~ 1‰) are smaller than in China (~ 2‰). The relatively muted δ^18^O response to millennial-scale events in the central IPWP has been noted before^20^ but it remains an open question as to how this translates into differences in the magnitude of monsoon strength or rainfall variability. This interpretation of speleothem δ^18^O is consistent with the GPM hypothesis of anti-phased hemispheric summer monsoon intensities due to meridional tropical rainbelt translation and/or a shift of rainfall from offshore to onshore, during northern hemisphere millennial-scale events.

There are two sections of the Flores speleothem δ^18^O record affected by the presence of aragonite or non-primary calcite. Between 19.5 and 17.1 kyr BP, which is approximately synchronous with the ECR, stalagmite LR06-C5 is composed of a mixture of calcite and aragonite. The increase in δ^18^O brought about by the variable concentration of aragonite in this section was proportionally corrected for in Ayliffe et al^23^ and we continue to use their correction. Stalagmite LR09-J1 is composed of non-primary calcite between 52.2 and 43.5 kyr BP (Supplementary Fig. S3). There is a negative isotopic offset here relative to the surrounding primary calcite, which indicates some isotopic overprinting by a diagenetic fluid. Therefore, the δ^18^O values across this interval of the record should be treated with caution. However, relative changes in δ^18^O within this interval can still be interpreted.

### Monsoon strength and precipitation amount

A key question is whether the orbital and millennial-scale changes in speleothem δ^18^O for Flores correspond to changes in local rainfall amount? The “amount effect” should not be assumed a priori, especially as precipitation amount and IPWP convection strength may be decoupled during the last glacial period^21^. For millennial-scale events, the Flores speleothem δ^18^O record is antiphase with non-speleothem δ^18^O records of past monsoon variability such as the sediment reflectance record for the Arabian sea^42^ (Fig. 3). Previous interpretations of speleothem δ^18^O at Flores have invoked the amount effect^17,23^. However, analysis of Mg/Ca, a proxy for prior calcite precipitation which is modulated by local epikarst infiltration, show different responses at different timescales. Synchronous changes in speleothem δ^18^O and Mg/Ca over the deglaciation and Holocene^43^ indicate some influence of the amount effect on speleothem δ^18^O. However, on shorter timescales such as the last 2000 years, Mg/Ca and δ^18^O are decoupled^14^. Elsewhere stalagmite δ^13^C records from Australia have suggested Heinrich events in the IASM were wet^29^.

New evidence from the pattern of speleothem growth phases at Liang Luar indicates that most Heinrich events correlate with wet events on Flores (Table 1). Eleven speleothem growth phases start during Heinrich events compared to just four growth terminations. New growth phases include the YD (LR06-B1iii); H1 stadial (LR09-N1i); H1 full (LR11-K5ii, LR06-C6iii); H2 (LR09-E7ii, LR06-C6iv, LR06-C3i, LR06-B3ii and LR09-L1i); H5 (LR09-G7ii), and H5a (LR09-J1i). In contrast, LR11-K5ii, LR09-E7ii, and LR09-G4ii terminate during millennial-scale events, as does LR07-E1ii, but we believe this to be a special case (see below). Overall, the evidence is supportive, but not conclusive of a negative correlation between precipitation amount and speleothem δ^18^O at the millennial-scale, and therefore a wet response to most Heinrich events.

**Table 1:**
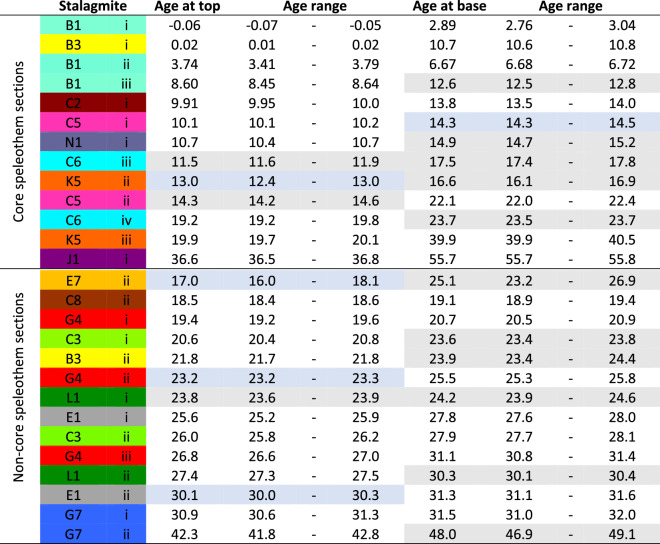
Summary of Liang Luar speleothem growth phases and their relationship with millennial-scale events.

Grey boxes indicate sections with new growth during millennial-scale ‘wet’ events or stop growing at the end of millennial-scale wet events or the ensuing 500 years. Blue boxes indicate sections that stop growing during millennial-scale ‘wet’ events or start growing at the end of millennial-scale ‘wet’ events or the ensuing 500 years. The best correlation of the ISCAM age model is not centered within the age range and, rarely, can be outside this range (see “Methods”).

### Variable responses to Heinrich events

**Figure 4:**
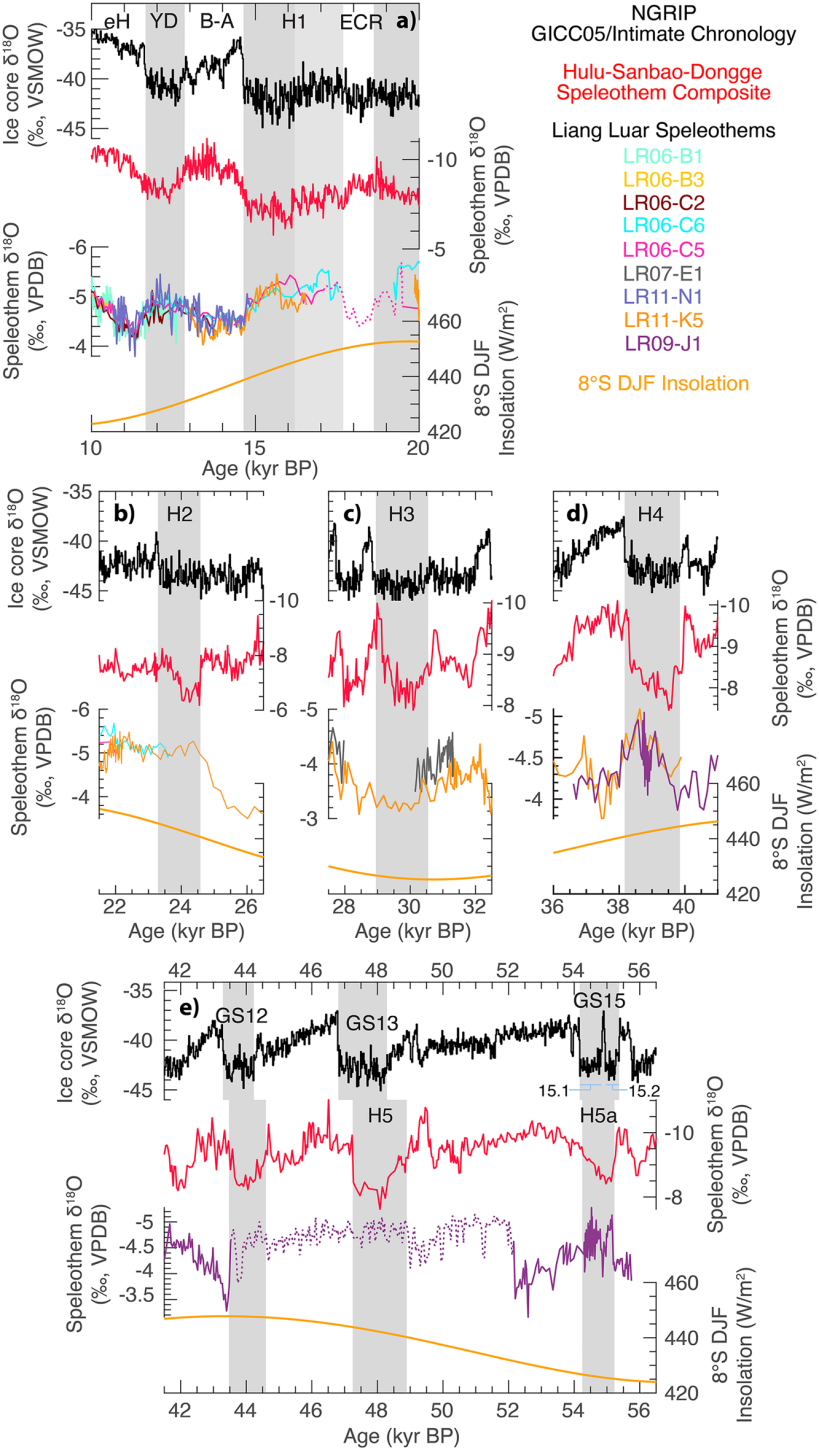
Isotopic responses to Heinrich events. Top to bottom in each panel: NGRIP ice core δ^18^O (black^47^), composite speleothem δ^18^O for China (red^34^), individual speleothem δ^18^O records for Flores (colours as shown in legend) and southern summer insolation (JJA) at 8°S (orange). Dotted lines indicate presence of aragonite or non-primary calcite mineralogies.

The lack of consistency in both magnitude and timing of the isotopic excursions at Heinrich events agrees with previous lower resolution or discontinuous records^25,30,31^. Examples of inconsistencies include no response to (or even a positive δ^18^O anomaly) at H3, a lack of definitive positive δ^18^O anomalies at the end of H2 and H5, and both large (> 1.5‰ at H2) and small (< 0.5‰ at H3 and H5) isotopic responses (Fig. 4). This suggests additional mechanisms, beyond meridional tropical rainbelt translation, influence Liang Luar speleothem δ^18^O during Heinrich events.

The onset of Heinrich event 2 (H2) recorded by stalagmite LR11-K5 coincides with the largest δ^18^O anomaly in the Flores speleothem record: −1.6‰ between 25.4 and 24.4 kyr BP (Fig. 4). Large positive isotope excursions occur in the Hulu and Borneo speleothem records at 24.6 kyr BP and 25.4 to 24.4 kyr BP, respectively. The longer duration and earlier onset of the isotopic excursion at Flores are likely due to age model effects which tend to force growth rate changes at U/Th age determinations. The magnitude of the δ^18^O anomaly in LR11-K5 is substantially larger than the typical 0.5–1.0‰ change observed at other Heinrich events. Contemporaneous stalagmite LR09-G4 provides replication of the negative δ^18^O excursion, but with a magnitude of 1.0‰ (Supplementary Fig. S4). Additional evidence for wetter conditions includes the onset of growth in five stalagmites during H2: LR09-E7 at 24.6 kyr BP, LR09-L1 at 23.9 kyr BP, LR06-B3 at 23.9 kyr BP, LR06-C3 at 23.7 kyr BP and delayed onset of growth in LR06-C6 at 23.6 kyr BP. This suggests a substantial increase in water availability did occur at Liang Luar at H2.

At the end of H2, the speleothem δ^18^O record does not return to higher pre-excursion values. The continuation of low δ^18^O beyond H2 may be related to orbital-scale forcing of increased rainfall from high southern summer insolation, the potential effect of increased continentality on rainfall δ^18^O, and/or source moisture changes, as discussed above.

Heinrich event 3 (H3) was previously described by Lewis et al.^32^ as a hiatus in Liang Luar stalagmite LR07-E1 between 30.1 and 27.8 kyr BP. LR11-K5 likely grew continuously through this period and contains a δ^18^O maximum between 30.2 and 28.3 kyr BP. However, the maximum is part of a broader 36–30 kyr BP increase in δ^18^O and decrease from 29 to 27 kyr BP. The broad δ^18^O maximum may be associated with the coincident southern summer insolation minimum. Abrupt isotopic changes at 30.2 kyr BP (positive) and 28.3 kyr BP (negative) are close to, but lag slightly, the onset and termination of Greenland Stadial (GS) 5.1 at 30.55 and 28.85 kyr BP. If H3 is recorded in the Flores δ^18^O record at all it is only weakly expressed and occurs as a positive δ^18^O anomaly.

Heinrich event 4 (H4) shows perhaps the clearest signal in the Liang Luar δ^18^O record. Stalagmites LR09-J1 and LR11-K5 both show negative δ^18^O excursions beginning at 39.5 kyr BP and ending at 38.1 kyr BP, synchronous with positive δ^18^O excursions in the speleothem records for China^34,35^ and Borneo^44^, a positive excursion in the Arabian sea monsoon records^42^ , and a negative δ^18^O anomaly at Cueva del Diamante and El Condor Caves^40^ (Fig. 3).

There has been discussion over which isotopic/climatic excursion in Greenland ice-core and terrestrial paleoclimate records corresponds to Heinrich event 5 (H5) in the north Atlantic sedimentary record. Sediment core records^45^ previously placed H5 at GS12. The original Hulu cave speleothem record^35^ hypothesised two locations: GS12 and GS13, preferring GS12 and assigning H5a to GS13 (since corrected). The Borneo speleothem record has positive δ^18^O excursions at both GS12 and GS13. GS13 was preferred as the location of H5 because it was the larger of the two δ^18^O anomalies^44^. A recent ice core study^46^ favours GS13 as H5 and have suggested GS15.1 as H5a. We note that precise synchronicity of north Atlantic sedimentary Heinrich events, Greenland stadials and tropical isotope excursions remains an ongoing area of research, largely due to chronological uncertainty.

At Liang Luar, stalagmite LR09-J1 shows negative δ^18^O excursions beginning at 55.2, 49.1 and 44.6 kyr BP (Fig. 4). All three are antiphase to the China and Borneo speleothem records and approximately correspond to GS15.2, 13 and 12. The latter two excursions begin earlier than the GICC05/INTIMATE ice-core chronology^47^ but are consistent with the Hulu speleothem record timing. The LR09-J1 δ^18^O response at GS15.2 is much more pronounced than at GS13 and 12 and corresponds to a period of faster speleothem growth. The growth rate is sufficiently rapid to recognise Greenland Interstadial (GI) 15.1 as a positive δ^18^O excursion between two negative δ^18^O excursions corresponding to GS15.2 and GS15.1. GI15.1 is not resolved in the composite speleothem record for China. However, given the recognised discrepancy between the China composite and the GICC05 chronology during MIS3^48^, we should consider that North Atlantic H5a begins at GS15.1. If so, then the similar magnitude δ^18^O anomalies observed at Flores for both GS15.1 and GS15.2 make the Flores H5a response (GS15.1) essentially indistinguishable from a ‘regular’ non-Heinrich event Greenland Stadial (GS15.2). To sum up, we interpret GS13 as H5 and GS15 as H5a (A GS15.1 vs. GS15.2 onset remains an open question) with the timing largely consistent between China and Flores.

We note that the δ^18^O response in LR09-J1 between 52 and 44 kyr BP, including GS13 (H5) and GS12, is somewhat muted due to diagenetic alteration. The artefact is related to a section of recrystallised calcite between two abrupt textural changes at 281 mm and 679 mm below the top of the stalagmite. High U concentrations^49^, contraction cracks and positively offset δ^13^C indicate this part of the stalagmite was originally deposited as aragonite (Supplementary Table S3, Supplementary Fig. S3). ISCAM indicates no apparent hiatuses or even growth rate slowdown across the textural changes; section age models overlap if the lower change is treated as a hiatus. An influence of (meteoric) diagenetic fluid on δ^18^O during recrystallisation might explain the flatness of the δ^18^O record during this period and therefore a muted response to H5.

### Orbital modulation of millennial-scale response

Orbital configuration may also play a role in modulating the response of Flores speleothem δ^18^O to Heinrich events. The muted response at H3 during low southern summer insolation and the large and extended response at H2 during rising southern summer insolation are cases in point. Krause et al.^20^ hypothesised that the absence of strong millennial-scale variability during the last glacial in stalagmites from southern Sulawesi could be caused by the proximity of Sulawesi (~ 400 km north of Flores) to the interhemispheric hinge point for seasonal changes in insolation. As low frequency, orbital-scale forcing changes the configuration of the tropical rainbelt, the response to superimposed high frequency forcing may change, producing non-identical responses when measured at a single paleoclimate site.

At Liang Luar, H3 shows, at most, a muted positive δ^18^O excursion between 30.2 and 28.3 kyr BP, and potentially no distinct isotopic response at all. Instead, the stalagmite δ^18^O record between 32 and 28 kyr BP is dominated by an extended δ^18^O maximum associated with the coincident summer insolation minimum. We hypothesise that the orbital state at this time led to an extreme ‘northward configuration’ of the tropical rainbelt. As a result, the southward cross-equatorial ‘push’ on the tropical rainbelt during H3 may not have been sufficient to cause a major isotopic/rainfall anomaly at low southern latitudes around Flores.

On the other hand, H2 occurred during a period of increasing tropical southern hemisphere summer insolation and at Liang Luar lacks an obvious positive δ^18^O anomaly at its termination. If a Heinrich event causes a temporary increase in monsoon strength through a more ‘southward configuration’ of the tropical rainbelt, then by its termination, the low frequency orbital forcing will have also pushed the rainbelt further south, such that there is little isotopic response to the end of the Heinrich event forcing. This phenomenon has been documented in the southern hemisphere tropics before, notably at the Salar del Uyuni in tropical South America^50^. However, unlike the Salar del Uyuni, we note that the inverse is not necessarily true at Liang Luar. H1 and H4 occur during periods of falling southern summer insolation, but the shifts in δ^18^O at the onsets and terminations of both Heinrich events are approximately the same magnitude.

Freshwater hosing experiments in climate models consistently put Flores near the hinge of wet/dry precipitation anomalies^31,32,51^, but the majority of hosing experiments occur under constant boundary conditions. Mohtadi et al.^51^ found similar spatial patterns when comparing hosing experiments at 21 and 38 kyr BP boundary conditions (i.e., H2 and H4). However, both are periods of relatively high southern summer insolation. The impact of different orbital configurations on the tropical hydroclimate response to Heinrich events, particularly in transient model simulations, requires further study.

## Conclusion

Three new stalagmites from Liang Luar extend the δ^18^O record back to 55.7 kyr BP. A further five new speleothems provide replication. We interpret the δ^18^O record as a proxy for the strength of the Indonesian–Australian Summer Monsoon, likely related to movements of the tropical rainbelt (either north–south and/or offshore-onshore). Speleothem growth phases support the idea that δ^18^O variability is related to local rainfall amount. To the first order, δ^18^O variability at Liang Luar is usually, but not always, consistent with antiphase behaviour of the Indonesian–Australian Summer Monsoon and the East Asian Summer Monsoon at both precession- and millennial- scales during the last glacial period. Negative δ^18^O excursions during Heinrich events and positive δ^18^O excursions during Dansgaard-Oeschger interstadials are often, but not always, present in the Liang Luar record. These results provide the continuous, first high-resolution, precisely dated, direct support for paired northern and southern hemisphere monsoon antiphase behaviour at the scale of millennial climate variability during the last glacial.

However, there are notable exceptions that are due, at least in part, to the idiosyncratic nature of individual speleothem drip hydrology. However, the extended record for Liang Luar shows that glacial-interglacial changes in insolation, ice-sheet extent and/or shelf exposure, influence orbital-scale monsoon variability, and initiate differences in the magnitude, onset, and termination of the monsoon response to millennial-scale events. We hypothesise that the role of orbital-scale configuration in propagating millennial-scale climate signals across the hemispheres may have been more important than previously recognised.

## Methods

### ^230^Th dating

A total of 73 new U-Th ages (Supplementary Tables 1–5) were produced for this study using multi-collector inductively coupled plasma mass spectrometry techniques^52,53^ at the University of Melbourne, the University of Minnesota and the University of Queensland. New U-Th ages were calculated, and published U-Th ages recalculated, using the Cheng et al.^52^ decay constants of 9.1705 × 10^–6^ for ^230^Th and 2.82206 × 10^–6^ for ^234^U, and put on the kyr BP timescale (where present is 1950CE). To correct for detrital thorium contamination an initial [^230^Th/^232^Th] ratio of 7 ± 2 was used. This value was determined by stratigraphic constraints^54^ in previous studies at Liang Luar^17,23^, and the new ages here do not provide any further constraint.

### Stable isotopic analysis

Powders for stable isotope analysis were milled from central slabs of collected speleothems at approximately 50-year resolution. A total of 1644 new stable isotope measurements were conducted at The Australian National University using Finnigan MAT-251 and Thermo MAT-253 isotope ratio mass spectrometers coupled to Kiel microcarbonate preparation devices. To ensure consistency among runs, in-run measurements of the NBS-19 standard (δ^18^O = −2.20‰ VPDB, n = 685) were complemented by less frequent measurements of NBS-18 (δ^18^O = −23.0‰ VPDB). We report a δ^18^O 2σ error of ± 0.09‰, which is the average of the 2σ errors for the NBS-19 standards in each mass spectrometer run (n = 131).

### Core and non-core stalagmites

To facilitate the production of an accurate composite record, we chose to focus on the highest quality, long duration, stalagmite sections (see Supplementary Discussion). Thirteen high quality sections were chosen: LR06-B1i, LR06-B1ii, LR06-B1iii, LR06-B3i, LR06-C2i, LR06-C5i, LR06-C5ii, LR09-N1ii, LR06-C6iii, LR06-C6iv, LR11-K5ii, LR11-K5iii and LR09-J1i. These are referred to as the ‘core speleothem’ sections (Supplementary Tables S1–S3, Figs. S1–S3). Fourteen speleothem sections, known as ‘non-core speleothem sections’, were not used in the production of the composite: LR06-B3ii, LR06-C3i, LR06-C3ii, LR11-C8ii, LR07-E1i, LR07-E1ii, LR09-E7ii, LR09-G4i, LR09-G4ii, LR09-G4iii, LR11-G7i, LR11-G7ii, LR09-L1i, LR09-L1ii, (Supplementary Tables S4–S5, Figs. S4–S8). An additional five sections contain insufficient U-Th or stable isotope measurements to provide useful results (LR11-C8i, LR09-E7i, LR09-G4iv, LR11-K5i, LR09-N1ii). Results and age models for the non-core stalagmites are discussed in the Supplementary Discussion. Isotopic results and discussion are primarily limited to the thirteen core stalagmite sections, with the growth phases of non-core stalagmites contributing supporting evidence.

### Age model construction

New age models were constructed for all core speleothem sections (both new and previously published) using ISCAM^55^ (Supplementary Discussion, Fig. S9). ISCAM uses a Monte-Carlo iterative correlation procedure. Initially, pointwise linear interpolated age models are produced for each core speleothem section. Then, individual section age models are iteratively adjusted to achieve maximum correlation between the δ^18^O records of contemporaneous stalagmites, while keeping the age model within the 2σ uncertainty bounds of the initial linear models. At the age-determination depths, the resulting age models are within the 1σ analytical uncertainty bound 94 out of 136 times (69%) and are always within the 2σ analytical uncertainty estimates. In this way, the U-Th ages of each stalagmite are used to refine the age model of all contemporaneous stalagmites. ISCAM also contains the option to align the δ^18^O values in overlapping sections. We consider this overfitting and therefore did not utilise this option. The stalagmite δ^18^O results presented remain those measured.

### Composite record production

A 50-year regularly spaced composite record was produced in MATLAB using the ISCAM-derived age models for the core stalagmite sections. The δ^18^O time series for individual sections were interpolated to one year resolution using a piecewise cubic Hermite interpolating spline. This creates a weighted average throughout each 50-year bin that ensures that anomalous points are not over- or under-represented; areas of low resolution have full data coverage, and areas of high resolution are not over-represented in the composite record. The 1-year interpolated record is then averaged to the mid-point of each 50-year bin to create a universal timescale. The start and end values of each section were screened to avoid overly large excursions caused by extrapolation. Finally, all the individual records in each bin are averaged together (Supplementary Fig. S10).

### Land-ice isotopic adjustment

On orbital time-scales, whole ocean δ^18^O changes by ~ 1.05‰ due to changing global land ice volume^56^, which influences stalagmite δ^18^O. We account for the influence of global land ice volume on ocean δ^18^O by subtracting the modelled ice-volume component of whole ocean δ^18^O^56^ from the 50-year binned stalagmite δ^18^O values to produce a seawater corrected composite record.

### Analysis of speleothem growth phases

In the analysis of speleothem growth phases, ages are reported to 3 significant figures with ranges based on uncertainties in the linear age models. The best correlation of the ISCAM age model is not centered within this range and, rarely, can be outside this range. Agreement of stalagmite growth phases with ‘wet’ events occurs when speleothem sections start growing during millennial-scale ‘wet’ events, or when speleothems stop growing at the end of the millennial-scale ‘wet’ events or the ensuing 500 years (Table 1, grey shading). Disagreement occurs when speleothems stop growing during a millennial-scale ‘wet’ event or start growing within 500 years of the end of a millennial scale ‘wet’ event (Table 1, blue shading). Due to age uncertainty, a stalagmite section could meet both criteria e.g., when a stalagmite starts growing with overlap between both the Heinrich event and the 500 years that follow. Where this occurs, the ISCAM age decides the preferred result. Start and end dates of millennial scale ‘wet’ events are taken from the INTIMATE chronology^47^ and therefore more accurately describe Dansgaard-Oeschger stadials than Heinrich events, although an association is assumed widely in the literature. In addition, some widely recognised millennial-scale events do not have major tie-points in the INTIMATE chronology but are recognised here. For example: an alternative start/intensification of H1 at 16.2 kyr BP and the start of H2 at 24.6 kyr BP.

## Supplementary Information

Supplementary Information.

## Data Availability

Data have been archived at the NCEI-NOAA Paleoclimatology Database at https://www.ncdc.noaa.gov/paleo/study/36953, and have been submitted to the SISAL database.
