# Supplementary material for: Antiphase response of the Indonesian–Australian monsoon to millennial-scale events of the last glacial period
Source: Sci Rep. 2022 Nov 23;12:20214. doi: 10.1038/s41598-022-21843-8 (PMC9691635; doi:10.1038/s41598-022-21843-8)
Supplement: Supplementary file 1 — Supplementary Information. [file 41598_2022_21843_MOESM1_ESM.docx]

**Antiphase response of the Indonesian–Australian monsoon to millennial-scale events of the last glacial period**

**SUPPLEMENTARY INFORMATION**

Nick Scroxton^1,2*^, Michael K. Gagan^1,3,4^, Linda K. Ayliffe^1^, Wahyoe S. Hantoro^3,5^, John C. Hellstrom^6^, Hai Cheng^7,8^, R. Lawrence Edwards^9^, Jian-xin Zhao^4^, Bambang W. Suwargadi^5^, and Hamdi Rifai^10^

^1^ Research School of Earth Sciences, The Australian National University, Canberra, ACT 2601, Australia.

^2^ Department of Geography, Maynooth University, Maynooth, Co. Kildare, Ireland

^3^ School of Earth, Atmospheric and Life Sciences, University of Wollongong, Wollongong, NSW 2522, Australia.

^4^ School of Earth and Environmental Sciences, The University of Queensland, St. Lucia, QLD 4072, Australia.

^5^ Research Center for Geotechnology, Indonesian Institute of Sciences, Bandung 40135, Indonesia.

^6^ School of Earth Sciences, The University of Melbourne, Parkville, VIC 3010, Australia.

^7^ Institute of Global Environmental Change, Xi’an Jiatong University, Xi’an 710049, China.

^8^ State Key Laboratory of Loess and Quaternary Geology, Institute of Earth Environment, Chinese Academy of Sciences, Xi’an 710061, China.

^9^ Department of Earth and Environmental Sciences, University of Minnesota, Minneapolis, MN 55455, USA.

^10^ Department of Physics, Universitas Negeri Padang, Padang 25131, Indonesia.

* Corresponding author: Nick Scroxton ([nick.scroxton@mu.ie](mailto:nick.scroxton@mu.ie))

New Core Records:

**Tables S1-S3:** U-Th dates for stalagmites LR09-N1, LR11-K5 and LR09-J1

**Figures S1-S3:** Individual descriptions, photographs and age models for stalagmites LR09-N1, LR11-K5 and LR09-J1

New Non-Core Records:

**Table S4:** U-Th dates for non-core stalagmites LR11-C8, LR09-E7 and LR09-G4

**Table S5:** U-Th dates for non-core stalagmites LR11-G7 and LR09-L1

**Figures S4-S8:** Individual descriptions, photographs, and age models for non-core stalagmites LR09-G4, LR11-C8, LR11-G7, LR09-L1 and LR09-E7

The Composite Record:

**Supplementary Discussion:** Age model and composite record construction

**Figure S9:** ISCAM age models for previously published stalagmite records

**Figure S10:** Comparison of the Ayliffe et al., (2013) composite with the new composite

**Figure S11:** Replication of core stalagmite δ^18^O records during the deglaciation

**Figure S12:** Replication of core and non-core speleothem δ^18^O records during the Extrapolar Climate Reversal.

**Table S1:** U-Th dates for stalagmite LR09-N1

| **Sample** | **Lab** | **Year of** | **Depth** | **U** | **[^230^Th/** | **±2σ** | **δ^234^U** | **±2σ** | **[^230^Th/** | **±2σ** | **Uncorr. age** | **±2σ** | **Corr. age** | **±2σ** | **δ^234^U** | **±2σ** |
| --- | --- | --- | --- | --- | --- | --- | --- | --- | --- | --- | --- | --- | --- | --- | --- | --- |
|  |  | **chemistry** | **(mm)** | **(ng g^-1^)** | **^238^U]** |  | **(measured)** |  | **^232^Th]** |  | **(kyr)** |  | **(kyr BP)** |  | **(initial)** |  |
| **LR09-N1** |  |  |  |  |  |  |  |  |  |  |  |  |  |  |  |  |
| LR09-N1-U10 | UMelb | 2012 | 10 | 23 | 0.09515 | 0.00075 | 2.8 | 2.2 | 197 | 3 | 10.87 | 0.09 | 10.44 | 0.14 | 2.9 | 2.3 |
| LR09-N1-U20 | UMelb | 2012 | 220.25 | 954 | 0.10470 | 0.00067 | 28.2 | 2.4 | 211 | 10 | 11.70 | 0.09 | 11.27 | 0.14 | 29.2 | 2.5 |
| LR09-N1-4 | UMelb | 2011 | 240.5 |  | 0.10378 | 0.00087 | 20.1 | 2.0 | 383 | 4 | 11.69 | 0.11 | 11.43 | 0.12 | 20.8 | 2.0 |
| LR09-N1-U30 | UMelb | 2012 | 384.75 | 32 | 0.11018 | 0.00054 | 7.3 | 2.0 | 314 | 3 | 12.63 | 0.07 | 12.30 | 0.11 | 7.5 | 2.0 |
| LR09-N1-3 | UMelb | 2011 | 461 |  | 0.1154 | 0.0010 | 16.8 | 2.4 | 445 | 6 | 13.13 | 0.13 | 12.87 | 0.14 | 17.5 | 2.5 |
| LR09-N1-U40 | UMelb | 2012 | 600.75 | 1257 | 0.12353 | 0.00060 | 50.0 | 2.5 | 2655 | 183 | 13.636 | 0.081 | 13.540 | 0.082 | 52.0 | 2.5 |
| LR09-N1-U50 | UMelb | 2012 | 760.25 | 240 | 0.1289 | 0.0016 | 6.5 | 2.0 | 841 | 18 | 14.94 | 0.20 | 14.76 | 0.20 | 6.8 | 2.1 |
| LR09-N1-2 | UMelb | 2011 | 819.75 |  | 0.1542 | 0.0017 | 18.8 | 2.4 | 492 | 10 | 17.89 | 0.22 | 17.59 | 0.23 | 19.7 | 2.6 |
| LR09-N1-U60 | UMelb | 2012 | 877.75 | 700 | 0.1575 | 0.0014 | 27.6 | 2.4 | 567 | 10 | 18.13 | 0.18 | 17.86 | 0.19 | 29.0 | 2.6 |

BP = before present, where present is defined as 1950CE

**Table S2:** U-Th dates for stalagmite LR11-K5

| **Sample** | **Lab** | **Year of** | **Depth** | **U** | **[^230^Th/** | **±2σ** | **δ^234^U** | **±2σ** | **[^230^Th/** | **±2σ** | **Uncorr. age** | **±2σ** | **Corr. age** | **±2σ** | **δ^234^U** | **±2σ** |
| --- | --- | --- | --- | --- | --- | --- | --- | --- | --- | --- | --- | --- | --- | --- | --- | --- |
|  |  | **chemistry** | **(mm)** | **(ng g^-1^)** | **^238^U]** |  | **(measured)** |  | **^232^Th]** |  | **(kyr)** |  | **(kyr BP)** |  | **(initial)** |  |
| **LR11-K5** |  |  |  |  |  |  |  |  |  |  |  |  |  |  |  |  |
| LR11-K5-U05 | UMinn | 2013 | 9 | 62 | 0.0407 | 0.0012 | 68.0 | 1.4 | 50 | 2 | 4.23 | 0.13 | 3.58 | 0.21 | 68.7 | 1.4 |
| LR11-K5-U10 | UMelb | 2012 | 45 | 152 | 0.1275 | 0.0021 | 104.4 | 4.5 | 181 | 3 | 13.35 | 0.24 | 12.80 | 0.28 | 108.3 | 4.6 |
| LR11-K5-U13 | UMinn | 2013 | 90.5 | 214 | 0.13249 | 0.00043 | 98.4 | 1.5 | 404 | 9 | 13.991 | 0.056 | 13.701 | 0.086 | 102.3 | 1.5 |
| LR11-K5-U16 | UMinn | 2013 | 142 | 125 | 0.13601 | 0.00049 | 84.1 | 1.4 | 783 | 19 | 14.594 | 0.063 | 14.409 | 0.072 | 87.6 | 1.5 |
| LR11-K5-U20 | UMelb | 2012 | 242.5 | 149 | 0.1552 | 0.0027 | 83.8 | 4.3 | 215 | 4 | 16.82 | 0.33 | 16.25 | 0.36 | 87.8 | 4.5 |
| LR11-K5-U30 | UMinn | 2013 | 257 | 248 | 0.18533 | 0.00046 | 74.1 | 1.5 | 206 | 4 | 20.61 | 0.07 | 19.91 | 0.20 | 78.4 | 1.6 |
| LR11-K5-U40 | UMelb | 2012 | 347.5 | 134 | 0.1979 | 0.0022 | 75.5 | 4.4 | 84 | 1 | 22.12 | 0.29 | 20.39 | 0.56 | 80.0 | 4.6 |
| LR11-K5-M1 | UMinn | 2015 | 469 | 52 | 0.20026 | 0.00095 | 75.4 | 1.6 | 1270 | 39 | 22.42 | 0.13 | 22.24 | 0.13 | 80.3 | 1.7 |
| LR11-K5-U44 | UMinn | 2013 | 483 | 102 | 0.21122 | 0.00079 | 89.4 | 1.2 | 436 | 9 | 23.43 | 0.11 | 23.03 | 0.14 | 95.5 | 1.2 |
| LR11-K5-U45 | UMinn | 2013 | 495 | 96 | 0.23127 | 0.00077 | 90.4 | 1.5 | 110 | 2 | 25.91 | 0.11 | 24.37 | 0.44 | 96.9 | 1.6 |
| LR11-K5-M2 | UMinn | 2015 | 506 | 160 | 0.24477 | 0.00056 | 106.9 | 1.6 | 732 | 15 | 27.14 | 0.09 | 26.84 | 0.11 | 115.4 | 1.7 |
| LR11-K5-U50 | UMelb | 2012 | 533.5 | 110 | 0.2583 | 0.0025 | 93.5 | 4.3 | 8168 | 286 | 29.27 | 0.36 | 29.19 | 0.35 | 101.5 | 4.6 |
| LR11-K5-M3 | UMinn | 2015 | 576 | 138 | 0.26973 | 0.00070 | 78.2 | 2.0 | 143700 | >71,900* | 31.28 | 0.12 | 31.22 | 0.12 | 85.4 | 2.1 |
| LR11-K5-M4 | UMinn | 2015 | 689 | 137 | 0.28492 | 0.00077 | 82.5 | 1.5 | 555 | 11 | 33.17 | 0.13 | 32.75 | 0.16 | 90.5 | 1.7 |
| LR11-K5-U60 | UMelb | 2012 | 798.25 | 60 | 0.2938 | 0.0032 | 85.5 | 4.3 | 1487 | 18 | 34.27 | 0.47 | 34.07 | 0.47 | 94.2 | 4.7 |
| *LR11-K5-U70* | *UMelb* | *2012* | *810.25* | *97* | *0.281* | *0.012* | *63.3* | *6.8* | *348* | *15* | *33.4* | *1.65* | *32.7* | *1.7* | *69.4* | *7.4* |
| LR11-K5-M5 | UMinn | 2015 | 830 | 142 | 0.30888 | 0.00074 | 64.5 | 1.8 | 956 | 20 | 37.24 | 0.14 | 36.94 | 0.16 | 71.6 | 2.0 |
| LR11-K5-M6 | UMinn | 2015 | 875 | 81 | 0.32070 | 0.00092 | 67.3 | 1.4 | 430 | 9 | 38.81 | 0.16 | 38.21 | 0.22 | 75.0 | 1.6 |
| LR11-K5-U80 | UMinn | 2013 | 914 | 88 | 0.3346 | 0.0012 | 67.3 | 1.5 | 277 | 6 | 40.84 | 0.20 | 39.91 | 0.32 | 75.3 | 1.6 |

BP = before present, where present is defined as 1950CE

*Dates in italics were not included in the age model*

* ^232^Th close to analytical blank. Therefore, the ^230^Th/^232^Th error was estimated from blank correction uncertainty, not the analytical uncertainty

**Table S3:** U-Th dates for stalagmite LR09-J1

| **Sample** | **Lab** | **Year of** | **Depth** | **U** | **[^230^Th/** | **±2σ** | **δ^234^U** | **±2σ** | **[^230^Th/** | **±2σ** | **Uncorr. age** | **±2σ** | **Corr. age** | **±2σ** | **δ^234^U** | **±2σ** |
| --- | --- | --- | --- | --- | --- | --- | --- | --- | --- | --- | --- | --- | --- | --- | --- | --- |
|  |  | **chemistry** | **(mm)** | **(ng g^-1^)** | **^238^U]** |  | **(measured)** |  | **^232^Th]** |  | **(kyr)** |  | **(kyr BP)** |  | **(initial)** |  |
| **LR09-J1** |  |  |  |  |  |  |  |  |  |  |  |  |  |  |  |  |
| LR09-J1-5 | UMelb | 2011 | 11.5 |  | 0.3118 | 0.0014 | 98.3 | 2.4 | 5919 | 306 | 36.22 | 0.23 | 36.12 | 0.23 | 108.8 | 2.6 |
| LR09-J1-M1 | UMinn | 2015 | 15 | 357 | 0.31377 | 0.00061 | 95.1 | 1.7 | 127883 | 30947 | 36.62 | 0.12 | 36.56 | 0.12 | 105.4 | 1.9 |
| LR09-J1-M2 | UMinn | 2015 | 68 | 326 | 0.32501 | 0.00061 | 84.4 | 1.7 | 63161 | 13132 | 38.66 | 0.13 | 38.59 | 0.13 | 94.1 | 1.8 |
| LR09-J1-U10 | UMelb | 2012 | 147 | 225 | 0.3299 | 0.0013 | 87.6 | 1.5 | 10900 | 84 | 39.21 | 0.21 | 39.13 | 0.21 | 97.9 | 1.7 |
| LR09-J1-U25 | UMinn | 2013 | 194 | 1105 | 0.34656 | 0.00073 | 85.3 | 1.3 | 67043 | 4703 | 41.71 | 0.14 | 41.65 | 0.14 | 96.0 | 1.4 |
| *LR09-J1-M3* | *UMinn* | 2015 | *204.5* | *204* | *0.32691* | *0.00061* | *85.0* | *1.2* | *116566* | *56719* | *38.90* | 0.12 | *38.84* | *0.12* | *94.8* | *1.3* |
| LR09-J1-U30 | UMelb | 2012 | 292.5 | 1649 | 0.3553 | 0.0014 | 68.9 | 3.0 | 4718 | 27 | 43.85 | 0.27 | 43.74 | 0.27 | 78.0 | 3.4 |
| *LR09-J1-2* | *UMelb* | 2012 | *518.75* |  | *0.4139* | *0.0040* | *95.7* | *2.5* | *46869* | *9836* | *51.33* | 0.66 | *51.26* | *0.66* | *110.6* | *2.8* |
| LR09-J1-U50 | UMelb | 2012 | 554.5 | 4100 | 0.39749 | 0.00064 | 88.5 | 1.4 | 35894 | 13001 | 49.21 | 0.15 | 49.14 | 0.15 | 101.7 | 1.6 |
| *LR09-J1-M4* | *UMinn* | 2015 | *560* | *4253* | *0.4741* | *0.0025* | *96.3* | *2.5* | *117285* | *3234* | *61.17* | 0.48 | *61.10* | *0.48* | *114.5* | *2.9* |
| *LR09-J1-M5* | *UMinn* | 2015 | *685* | *7027* | *0.4938* | *0.0031* | *75.2* | *2.6* | *212682* | *5725* | *66.46* | 0.62 | *66.40* | *0.62* | *90.7* | *3.1* |
| LR09-J1-U70 | UMelb | 2012 | 696.25 | 176 | 0.4067 | 0.0014 | 56.9 | 1.6 | 5303 | 52 | 52.72 | 0.26 | 52.60 | 0.26 | 66.0 | 1.9 |
| LR09-J1-U80 | UMelb | 2012 | 728.5 | 26 | 0.4154 | 0.0014 | 53.2 | 1.3 | 5316 | 52 | 54.43 | 0.26 | 54.31 | 0.26 | 62.0 | 1.4 |
| LR09-J1-U90 | UMelb | 2012 | 909.8 | 885 | 0.4312 | 0.0016 | 87.3 | 2.3 | 6975 | 88 | 54.66 | 0.32 | 54.56 | 0.32 | 101.8 | 2.7 |
| LR09-J1-M6 | UMinn | 2015 | 968 | 337 | 0.42504 | 0.00073 | 64.6 | 1.4 | 48045 | 4789 | 55.24 | 0.18 | 55.17 | 0.18 | 75.5 | 1.7 |
| *LR09-J1-1* | *UMelb* | 2011 | *1021* |  | *0.4209* | *0.0017* | *62.7* | *2.3* | *4558* | *210* | *54.67* | 0.33 | *54.55* | *0.33* | *73.2* | *2.6* |

BP = before present, where present is defined as 1950CE

*Dates in italics were not included in the age model*

**Figures S1-S3:** Individual descriptions, photographs, and age models for core stalagmites LR09-N1, LR11-K5 and LR09-J1. Figures that follow include a photograph of each of the three core stalagmites (with scale bar) showing the locations of stable isotope sampling transects (blue lines) and samples for U-Th dating (red dots) Ages with 2σ errors are indicated in red text. Position of growth hiatuses are indicated with yellow arrows. The accompanying age-depth plot is based on U-Th ages with 2σ error (black bars). Shading shows the 2σ error of independent linearly interpolated age model. The best-fit of the final ISCAM age model is in black line. The resulting δ^18^O time-series (colour, solid line) is plotted for comparison with the core stalagmites covering the same time-interval (grey).


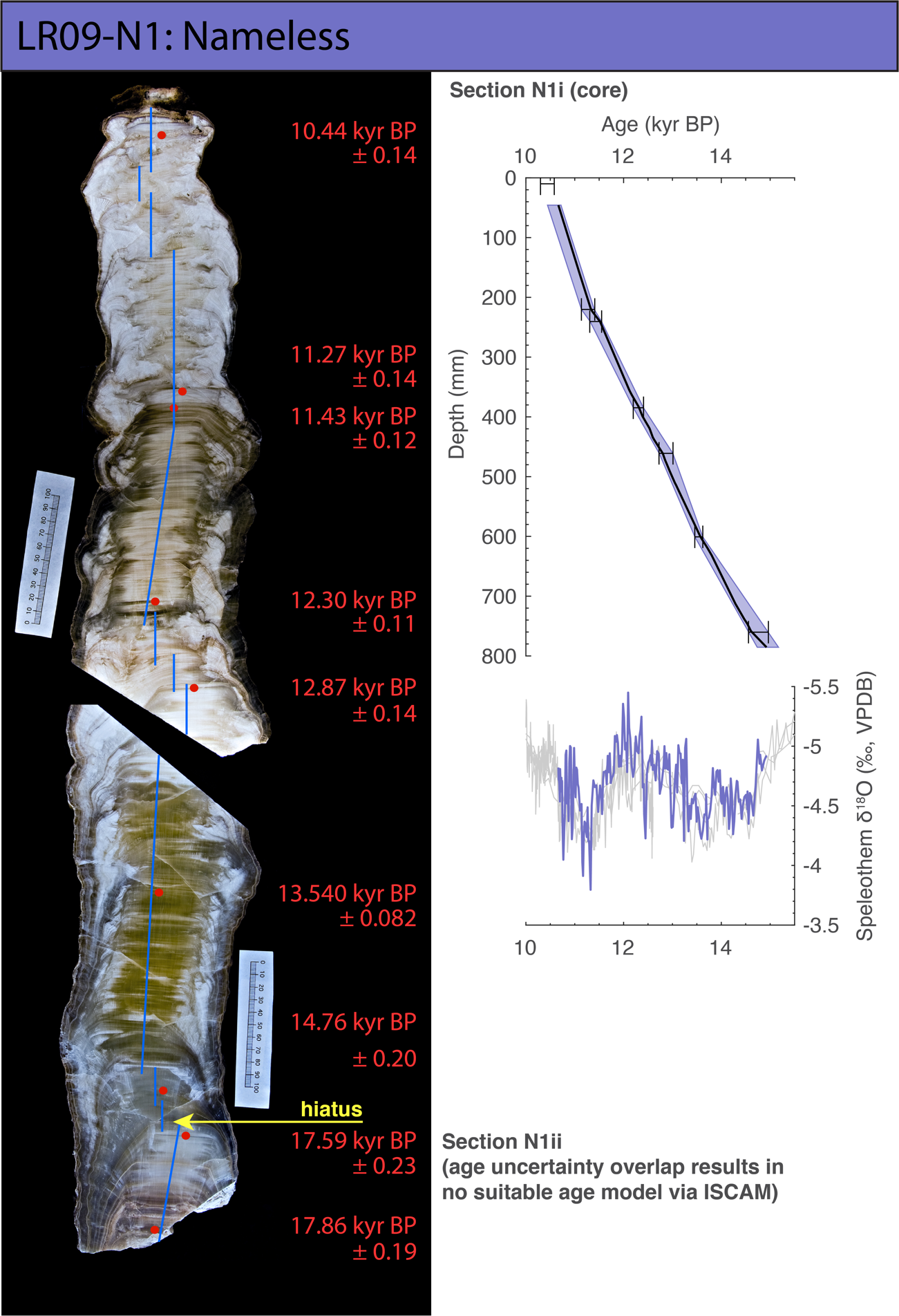


**Figure S1:** LR09-N1 is a relatively large diameter 90 cm tall thick candlestick-type stalagmite that grew mainly during the last deglaciation (~15–10 kyr BP). The bottom two-thirds are made of translucent calcite, with a wide flat top and visible fine laminae. The top section is similarly laminated but with milky opaque flanks.


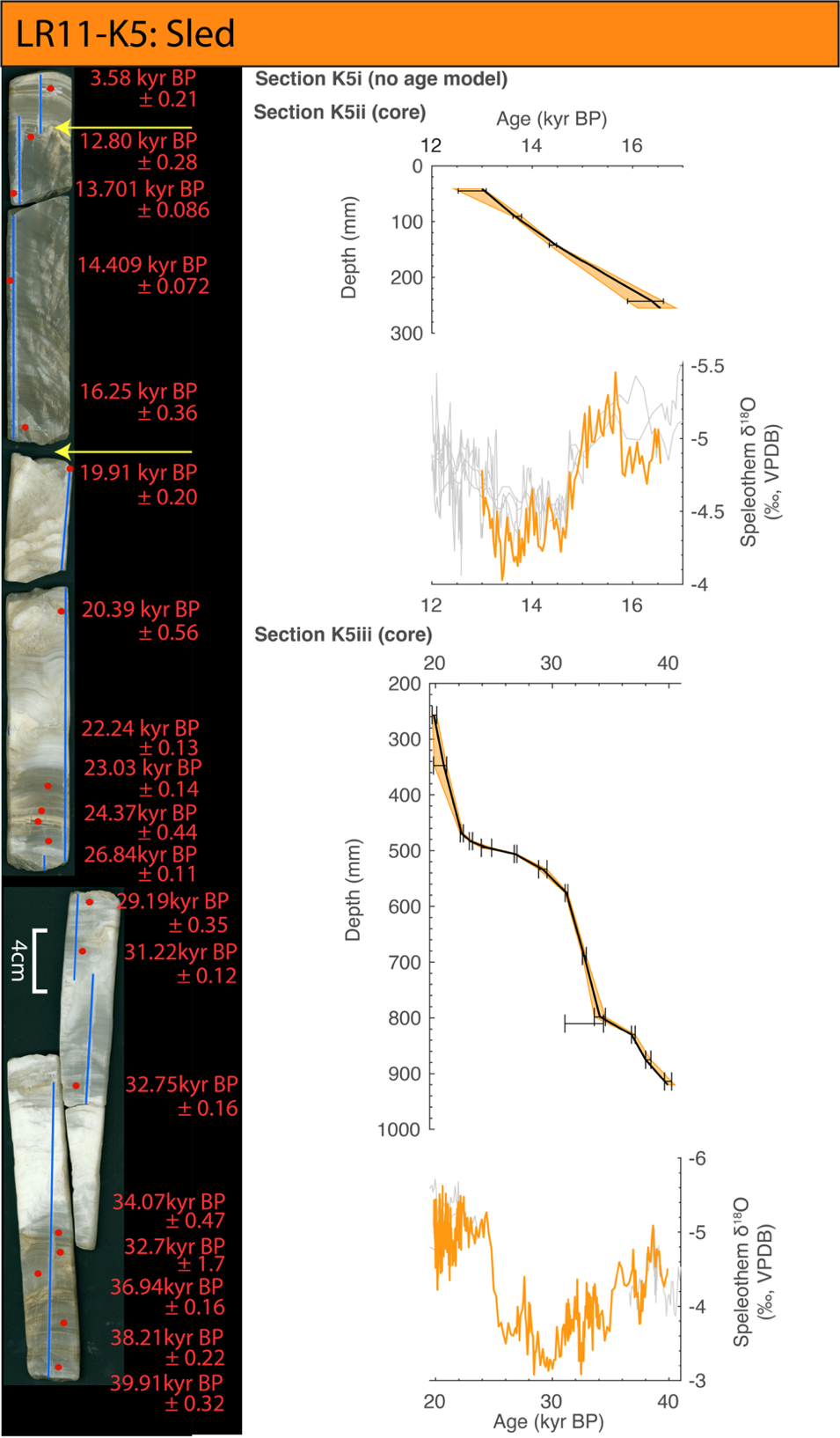


**Figure S2:** LR11-K5 is a 92 cm tall stalagmite, with a large diameter (~40 cm) and tapering shape that grew from ~40–12 kyr BP. It was too heavy for removal, so the stalagmite was cored inside the cave. It is difficult to assess the full nature of the stalagmite from cores alone, but it appears to be composed of dark translucent calcite with occasional laminae, along with some milky opaque sections during times of relatively fast growth. The uppermost Holocene section was analysed (K5i), but it has only one date and is of insufficient length to warrant inclusion in the composite record. The section between 20 and 16 kyr BP is missing, but this may be partly or wholly due to difficulties in recovering the full length of the stalagmite from drill cores rather than purely a hiatus in growth. A potential hiatus could span 29 to 24 kyr BP. However, running the stalagmite age model as a continuous, but slow growing, section achieved better correlation with the non-core records, particularly the large negative excursion in δ^18^O at 25 to 24.4 kyr BP. This 1.5‰ excursion coincides with a similar change in LR09-G4 at the onset of Heinrich event 2, and occurs over the course of 6 data points and 5mm of growth, al, suggesting that stalagmite growth was continuous through this period.


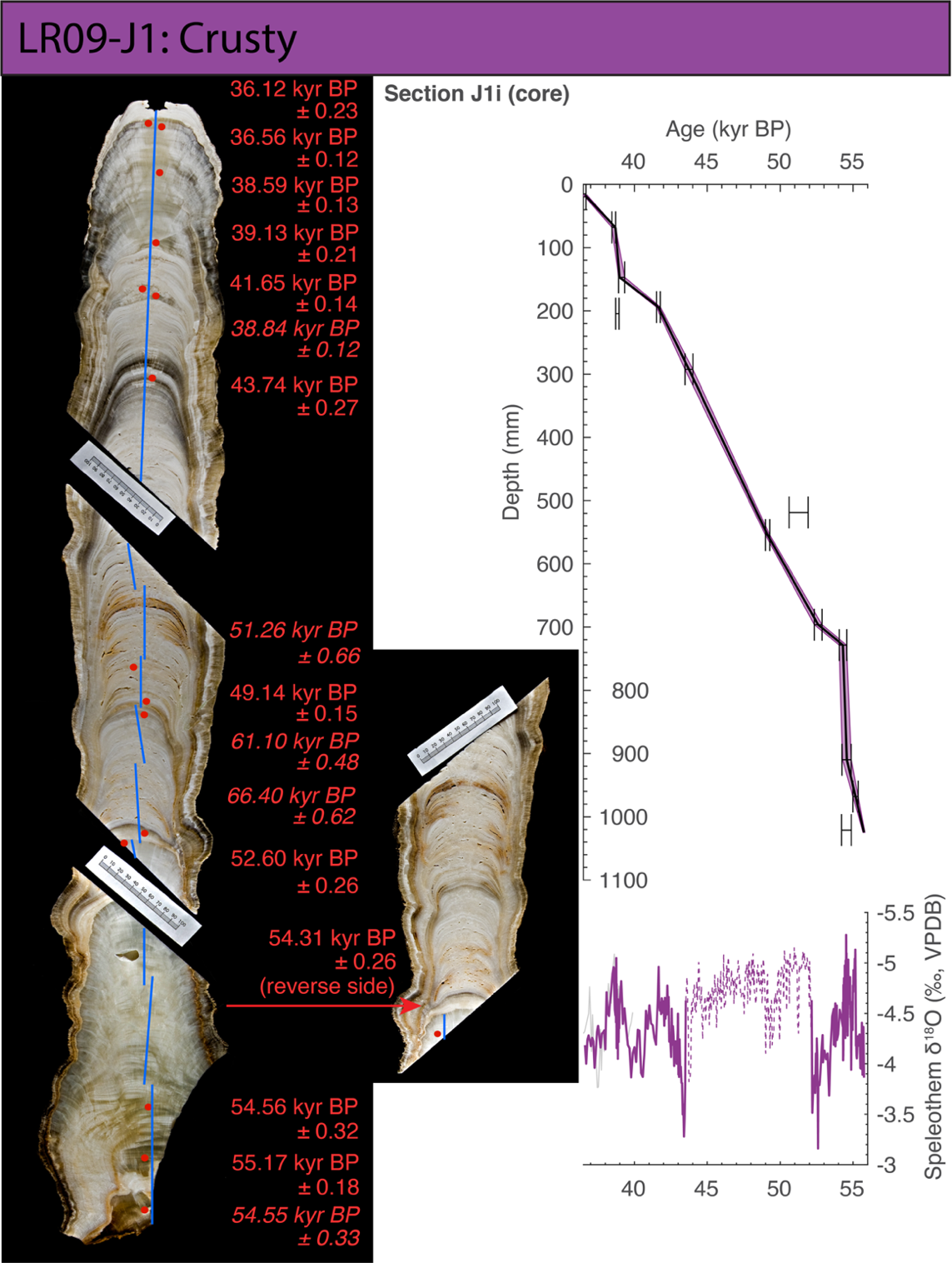


**Figure S3:** LR09-J1 is a 103 cm tall stalagmite with an irregular shape and crusty external texture created by a 1 cm thick fibrous calcite crust growing radially out from the stalagmite. It grew from ~55 to 36 kyr BP. The bottom 300 mm is a finely laminated, fast-growing, high-quality calcite section with a raised central growth axis. However, the middle section contains contraction cracks, indicating that this section may have been deposited as aragonite that later altered to calcite. This hypothesis is supported by uranium concentrations in excess of 1000ng/g in the central dating samples and by the δ^13^C signature, which is positively offset by approximately ~1.5‰ to 2‰ relative to the rest of the record. On the other hand, the δ^18^O for the aragonite section appears to be negatively offset. This suggests that there has been significant isotopic overprinting during the recrystallisation process for δ^18^O, but not for δ^13^C. As no adjustment can be properly quantified for the δ^18^O record, none is taken. The top 150 mm is high-quality calcite similar to the base of the stalagmite but with a growth rate comparable to the middle section. The stalagmite is topped by a small post-depositional dissolution cup. Four U-Th ages out of stratigraphic order were rejected in the construction of the age model. J1-M3 was rejected as it had lower uranium concentrations and an anomalously young age. J1-M4 and J1-M3 both have anomalously old ages and high ^230^Th/^232^Th. J1-1 was rejected as it has large age uncertainty and its location on the offcut rather than the slab limits the accuracy of its vertical position along the growth axis.

**Table S4:** U-Th dates for non-core stalagmites LR11-C8, LR09-E7 and LR09-G4

| **Sample** | **Lab** | **Year of** | **Depth** | **U** | **[^230^Th/** | **±2σ** | **δ^234^U** | **±2σ** | **[^230^Th/** | **±2σ** | **Uncorr. age** | **±2σ** | **Corr. age** | **±2σ** | **δ^234^U** | **±2σ** |
| --- | --- | --- | --- | --- | --- | --- | --- | --- | --- | --- | --- | --- | --- | --- | --- | --- |
|  |  | **chemistry** | **(mm)** | **(ng g^-1^)** | **^238^U]** |  | **(measured)** |  | **^232^Th]** |  | **(kyr)** |  | **(kyr BP)** |  | **(initial)** |  |
| **LR11-C8** |  |  |  |  |  |  |  |  |  |  |  |  |  |  |  |  |
| LR11-C8-2 | UMinn | 2011 | 0 | 179 | 0.10327 | 0.00049 | 236.0 | 2.0 | 38 | 1 | 9.49 | 0.05 | 7.76 | 0.49 | 241.3 | 2.1 |
| LR11-C8-U20 | UMelb | 2012 | 355 | 234 | 0.1345 | 0.0013 | 257.8 | 3.6 | 845 | 32 | 12.29 | 0.13 | 12.13 | 0.13 | 266.8 | 3.7 |
| LR11-C8-U40 | UMelb | 2012 | 627 | 230 | 0.15964 | 0.00099 | 264.2 | 2.7 | 1229 | 16 | 14.65 | 0.10 | 14.51 | 0.11 | 275.3 | 2.8 |
| LR11-C8-U50 | UMelb | 2012 | 658 | 302 | 0.1835 | 0.0011 | 162.7 | 3.3 | 3922 | 52 | 18.66 | 0.13 | 18.57 | 0.13 | 171.5 | 3.5 |
| LR11-C8-1 | UMelb | 2011 | 852 | 272 | 0.1915 | 0.0019 | 178.0 | 3.9 | 2468 | 152 | 19.26 | 0.22 | 19.15 | 0.22 | 188.0 | 4.1 |
| **LR09-E7** |  |  |  |  |  |  |  |  |  |  |  |  |  |  |  |  |
| LR09-E7-2 | UMelb | 2011 | 11 |  | 0.01840 | 0.00040 | 38.8 | 2.3 | 10 | 0 | 1.95 | 0.04 | 0.50 | 0.40 | 38.9 | 2.3 |
| LR09-E7-3 | UMelb | 2011 | 396 |  | 0.13480 | 0.00090 | 80.0 | 2.1 | 309 | 3 | 14.51 | 0.11 | 14.15 | 0.14 | 83.3 | 2.2 |
| LR09-E7-1 | UQ | 2010 | 974 |  | 0.18681 | 0.00075 | 80.4 | 1.0 | 36 | 0 | 20.7 | 0.10 | 16.9 | 1.1 | 84.3 | 1.1 |
| *LR09-E7-U29* | *UMelb* | 2011 | *1098* | *145* | *0.2090* | *0.0044* | *78.7* | *5.1* | *43* | *1* | *23.4* | 0.56 | *19.9* | *1.2* | *83.2* | *5.4* |
| LR09-E7-U35 | UMelb | 2012 | 1160 | 199 | 0.1900 | 0.0042 | 89.1 | 5.3 | 75 | 2 | 20.85 | 0.53 | 19.00 | 0.73 | 94.1 | 5.6 |
| LR09-E7-U50 | UMelb | 2012 | 1334.5 | 212 | 0.2495 | 0.0043 | 81.9 | 5.0 | 36 | 1 | 28.5 | 0.58 | 23.5 | 1.6 | 87.6 | 5.3 |
| **LR09-G4** |  |  |  |  |  |  |  |  |  |  |  |  |  |  |  |  |
| LR09-G4-2 | UMelb | 2011 | 24 |  | 0.1787 | 0.0016 | 73.4 | 2.8 | 8554 | 1198 | 19.82 | 0.21 | 19.74 | 0.21 | 77.6 | 3.0 |
| LR09-G4-3 | UMelb | 2011 | 79 |  | 0.1852 | 0.0017 | 76.3 | 2.4 | 8685 | 1375 | 20.54 | 0.21 | 20.47 | 0.21 | 80.9 | 2.5 |
| LR09-G4-4 | UMinn | 2011 | 111.5 | 223 | 0.20524 | 0.00051 | 54.4 | 1.9 | 21078 | 2036 | 23.561 | 0.088 | 23.493 | 0.088 | 58.2 | 2.0 |
| *LR09-G4-U20* | *UMelb* | 2010 | *155.5* | *127* | *0.22099* | *0.00091* | *50.2* | *2.3* | *2426* | *23* | *25.71* | 0.14 | *25.59* | *0.14* | *53.9* | *2.5* |
| LR09-G4-5 | UMelb | 2011 | 197.5 |  | 0.2211 | 0.0016 | 65.7 | 2.8 | 2229 | 170 | 25.29 | 0.22 | 25.16 | 0.22 | 70.6 | 3.0 |
| LR09-G4-6 | UMelb | 2011 | 227.25 |  | 0.2336 | 0.0015 | 59.0 | 2.2 | 7293 | 431 | 27.11 | 0.21 | 27.03 | 0.21 | 63.7 | 2.4 |
| LR09-G4-U30 | UMelb | 2012 | 299.75 | 222 | 0.24710 | 0.00048 | 54.4 | 1.2 | 5572 | 96 | 29.049 | 0.087 | 28.955 | 0.087 | 59.1 | 1.3 |
| LR09-G4-U40 | UMelb | 2012 | 485 | 202 | 0.26064 | 0.00097 | 49.0 | 2.1 | 3421 | 81 | 31.07 | 0.16 | 30.96 | 0.16 | 53.5 | 2.3 |
| LR09-G4-7 | UMelb | 2011 | 620.85 |  | 0.2653 | 0.0022 | 65.9 | 2.3 | 10102 | 1460 | 31.11 | 0.31 | 31.03 | 0.31 | 71.9 | 2.5 |
| LR09-G4-8 | UMinn | 2011 | 640.6 | 2997 | 0.3250 | 0.0013 | 66.0 | 2.2 | 77987 | 2256 | 39.50 | 0.23 | 39.43 | 0.23 | 73.8 | 2.5 |

BP = before present, where present is defined as 1950CE

*Dates in italics were not included in the age model*

**Table S5:** U-Th dates for non-core stalagmites LR11-G7 and LR09-L1

| **Sample** | **Lab** | **Year of** | **Depth** | **U** | **[^230^Th/** | **±2σ** | **δ^234^U** | **±2σ** | **[^230^Th/** | **±2σ** | **Uncorr. age** | **±2σ** | **Corr. age** | **±2σ** | **δ^234^U** | **±2σ** |
| --- | --- | --- | --- | --- | --- | --- | --- | --- | --- | --- | --- | --- | --- | --- | --- | --- |
|  |  | **chemistry** | **(mm)** | **(ng g^-1^)** | **^238^U]** |  | **(measured)** |  | **^232^Th]** |  | **(kyr)** |  | **(kyr BP)** |  | **(initial)** |  |
| **LR11-G7** |  |  |  |  |  |  |  |  |  |  |  |  |  |  |  |  |
| LR11-G7-2 | UMinn | 2011 | 57.5 | 205 | 0.28496 | 0.00071 | 109.3 | 1.6 | 201 | 4 | 32.21 | 0.12 | 31.18 | 0.30 | 119.4 | 1.7 |
| LR11-G7-U10 | UMelb | 2012 | 110.5 | 159 | 0.2791 | 0.0014 | 97.9 | 3.6 | 214 | 2 | 31.84 | 0.23 | 30.87 | 0.34 | 106.8 | 3.9 |
| LR11-G7-U31 | UMinn | 2013 | 416 | 134 | 0.29300 | 0.00076 | 108.8 | 1.5 | 113 | 2 | 33.28 | 0.12 | 31.45 | 0.53 | 119.0 | 1.6 |
| LR11-G7-U40 | UMelb | 2012 | 427 | 114 | 0.3644 | 0.0018 | 98.4 | 2.6 | 192 | 2 | 43.67 | 0.30 | 42.30 | 0.48 | 110.9 | 2.9 |
| LR11-G7-U60 | UMelb | 2012 | 942 | 188 | 0.3759 | 0.0013 | 97.2 | 2.7 | 1861 | 13 | 45.43 | 0.26 | 45.23 | 0.26 | 110.5 | 3.1 |
| LR11-G7-1 | UMelb | 2011 | 984.4 | 5017 | 0.3899 | 0.0052 | 105.1 | 3.0 | 2395 | 59 | 47.09 | 0.80 | 46.91 | 0.80 | 120.0 | 3.4 |
| **LR09-L1** |  |  |  |  |  |  |  |  |  |  |  |  |  |  |  |  |
| LR09-L1-U10 | UMinn | 2013 | 24 | 4135 | 0.22487 | 0.00043 | 143.5 | 1.4 | 2871 | 63 | 23.767 | 0.070 | 23.653 | 0.071 | 153.4 | 1.5 |
| LR09-L1-1 | UMelb | 2011 | 112 |  | 0.2317 | 0.0024 | 157.0 | 3.1 | 1893 | 174 | 24.25 | 0.29 | 24.11 | 0.29 | 168.1 | 3.2 |
| LR09-L1-U15 | UMinn | 2013 | 146 | 7383 | 0.25730 | 0.00043 | 149.0 | 1.4 | 19398 | 490 | 27.491 | 0.076 | 27.419 | 0.076 | 161.0 | 1.5 |
| LR09-L1-P | NONE | 2008 | 640.5 |  |  |  |  |  | 8447 |  |  |  | 29.86 | 0.13 |  |  |

BP = before present, where present is defined as 1950CE

* Full geochemical information for date LR09-L1-P cannot be located in the repository at any of the participating labs.

**Figure S4-S8:** Individual descriptions, photographs, and age models for non-core stalagmites LR09-G4, LR11-C8, LR11-G7, LR09-L1 and LR09-E7. Figures S4-S8 that follow include a photograph of each of the five non-core stalagmites (with scale bar) showing the locations of stable isotope sampling transects (blue lines) and samples for U-Th dating (red dots). Ages and 2σ errors are indicated in red text. Positions of growth hiatuses are indicated with yellow arrows. The accompanying age-depth plot is based on U-Th ages with 2σ errors (black bars). Shading shows the 2σ error of the independent linearly interpolated age model. The best-fit of the final ISCAM age model is in black. The resulting δ^18^O time-series (colour, solid line) is plotted for comparison with the core stalagmites covering the same time-interval (grey).


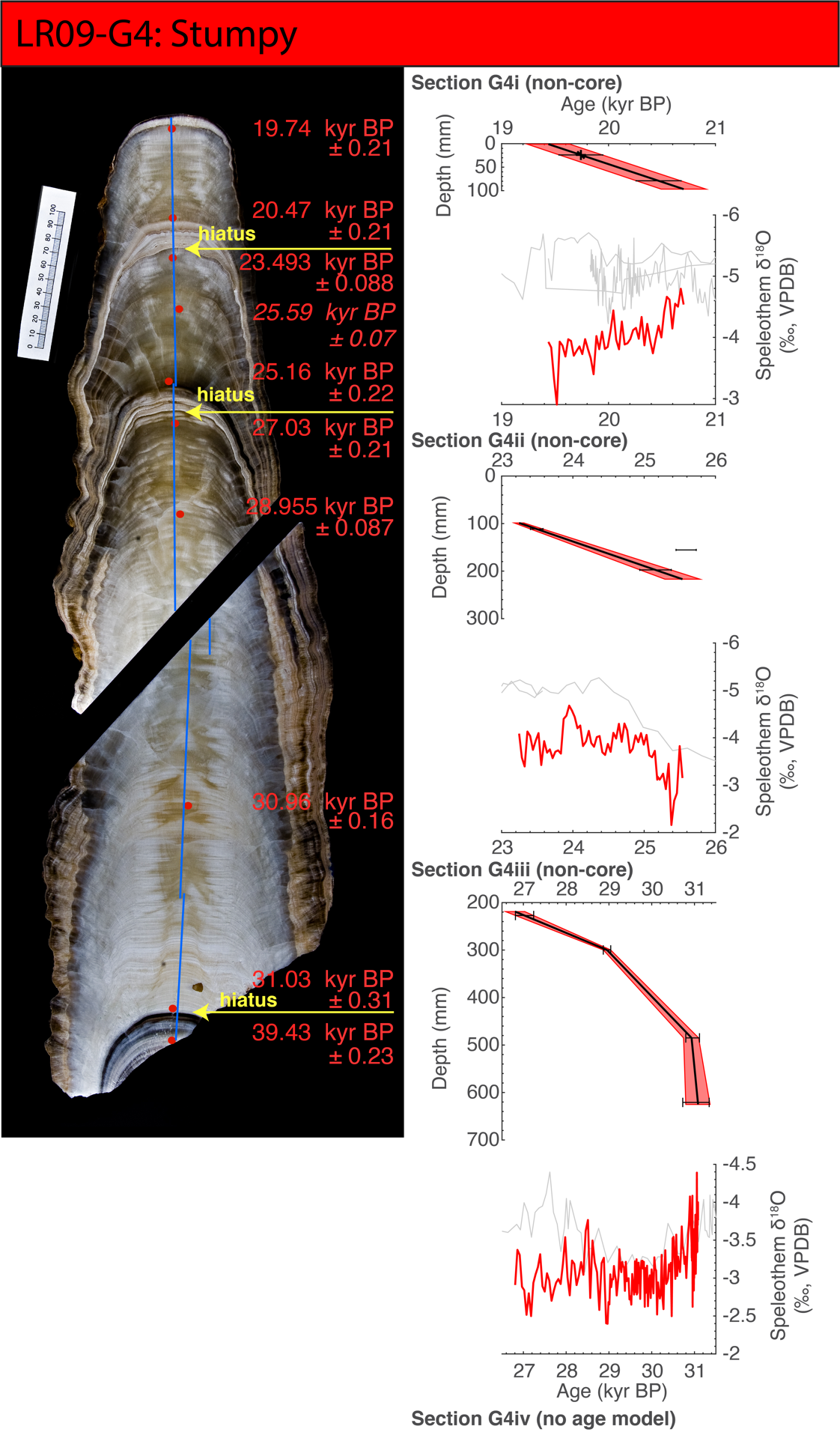


**Figure S4:** LR09-G4 is a 65 cm tall stalagmite with a conical shape that grew from ~31 to 27 kyr BP. It is composed of semi-translucent calcite with a faint slightly radial crystal fabric visible. The basal section provides good replication with LR09-J1 but is not included in the composite record as it has only seven data points and only one U-Th date, so growth rate cannot be constrained.


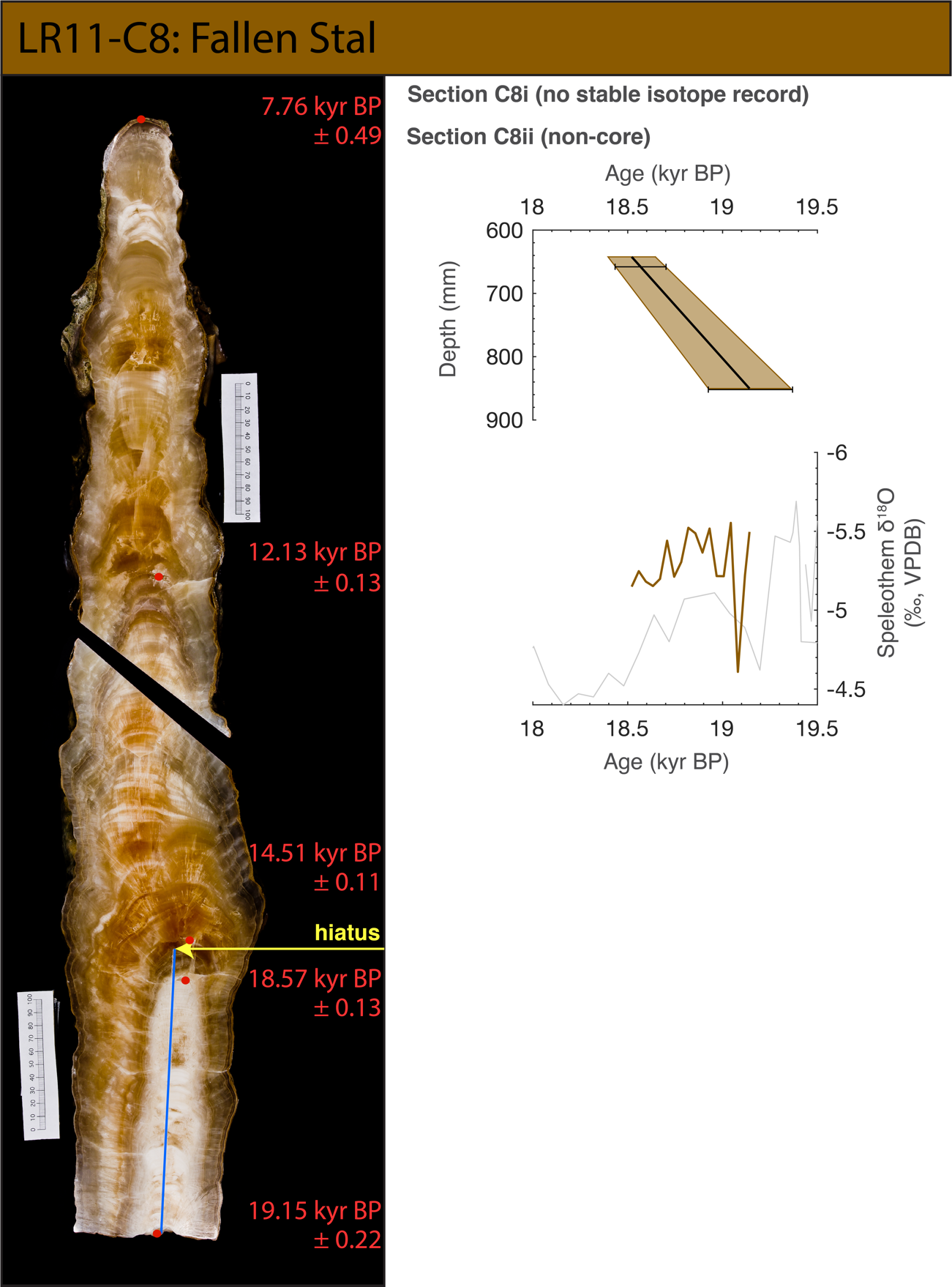


**Figure S5:** LR11-C8 is an 85 cm tall stalagmite with a fast-growing basal section that grew from ~19.2 to 18.5 kyr BP. The specimen had already fallen and was lightly cemented to a flowstone by a thin calcite crust. It is generally composed of orange, translucent calcite with visible laminae. The basal section is a candlestick stalagmite composed of milky calcite that spans the later part of the Last Glacial Maximum, but as the sequence is not particularly long it provides replication only. The top section covers the deglaciation, but it was not analysed as sufficient replication of this period is provided by other stalagmites analysed previously. The δ^18^O values for LR11-C8 are low relative to those for coeval stalagmites.


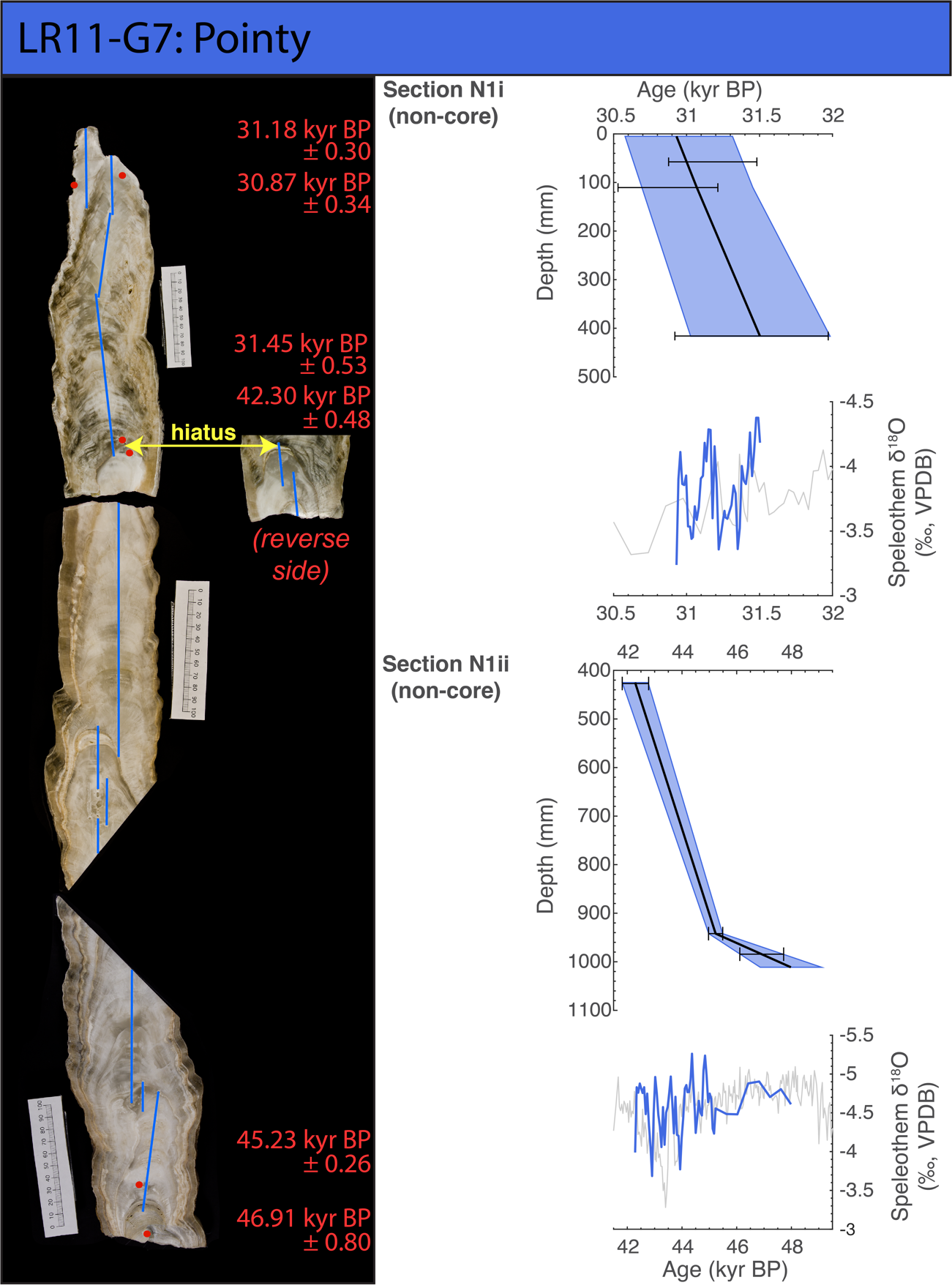


**Figure S6:** LR11-G7 is a 100 cm tall fast-growing stalagmite that grew from ~48 to 31 kyr BP with a clear hiatus between ~42 and 32 kyr BP. The growth axis has a large lateral movement across the slab, suggesting it may have been fed by different drips at different times. Consequently, the back face of the slab is also used to try to maintain the sampling transect along the growth axis. The bottom section appears to be a candle-type stalagmite, which has been overgrown by the wider top section. The very top has a finger-like protrusion, again suggesting a different drip source, but the ages and isotopes are continuous. Flat laminae in the top “finger” extend all the way to the edge of the stalagmite. It is likely that this stalagmite has been subject to some dissolution and may have been taller and/or wider in the past.

**
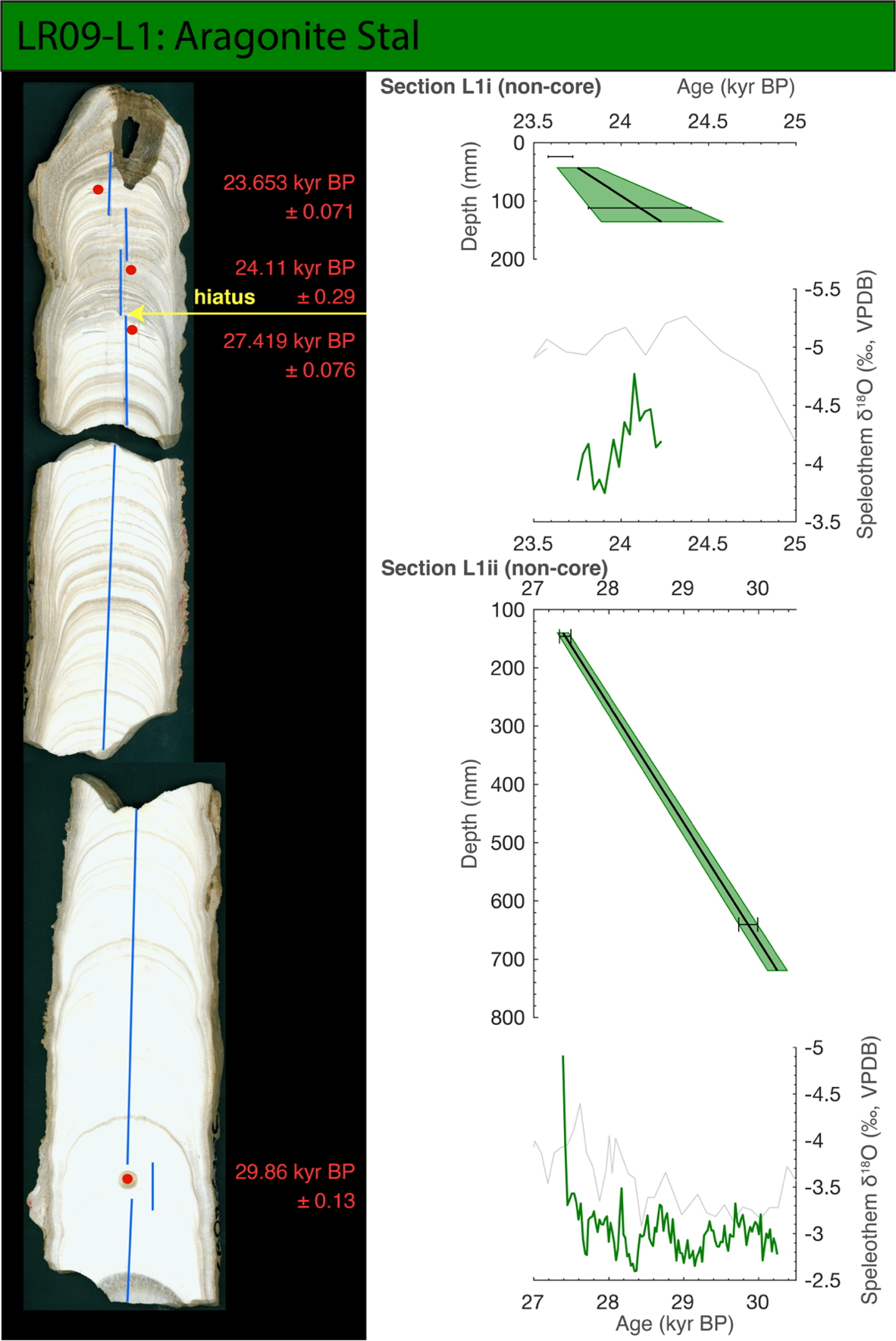
**

**Figure S7:** LR09-L1 is a 72 cm tall stalagmite that grew from ~30 to 23.5 kyr BP with a hiatus between ~27 and 24.3 kyr BP. The specimen is composed almost entirely of smooth, white, opaque aragonite. There are occasional laminae caused by mud layers and dirty dark calcite, which also forms the base. The top section is increasingly calcite rich but is of insufficient length to be included in the composite record. It is not certain whether this calcite is original or recrystallised aragonite. However, the δ^18^O of LR09-L1 is offset (to higher values) from the rest of the composite record by an average of 1.0‰ in the top section and 0.6‰ in the bottom section. The average offset is close to the positive offset for aragonite of 0.8‰ (relative to calcite) predicted by laboratory experiments and theoretical calculations at 25˚C (Tarutani et al., 1969; Kim et al., 2007).


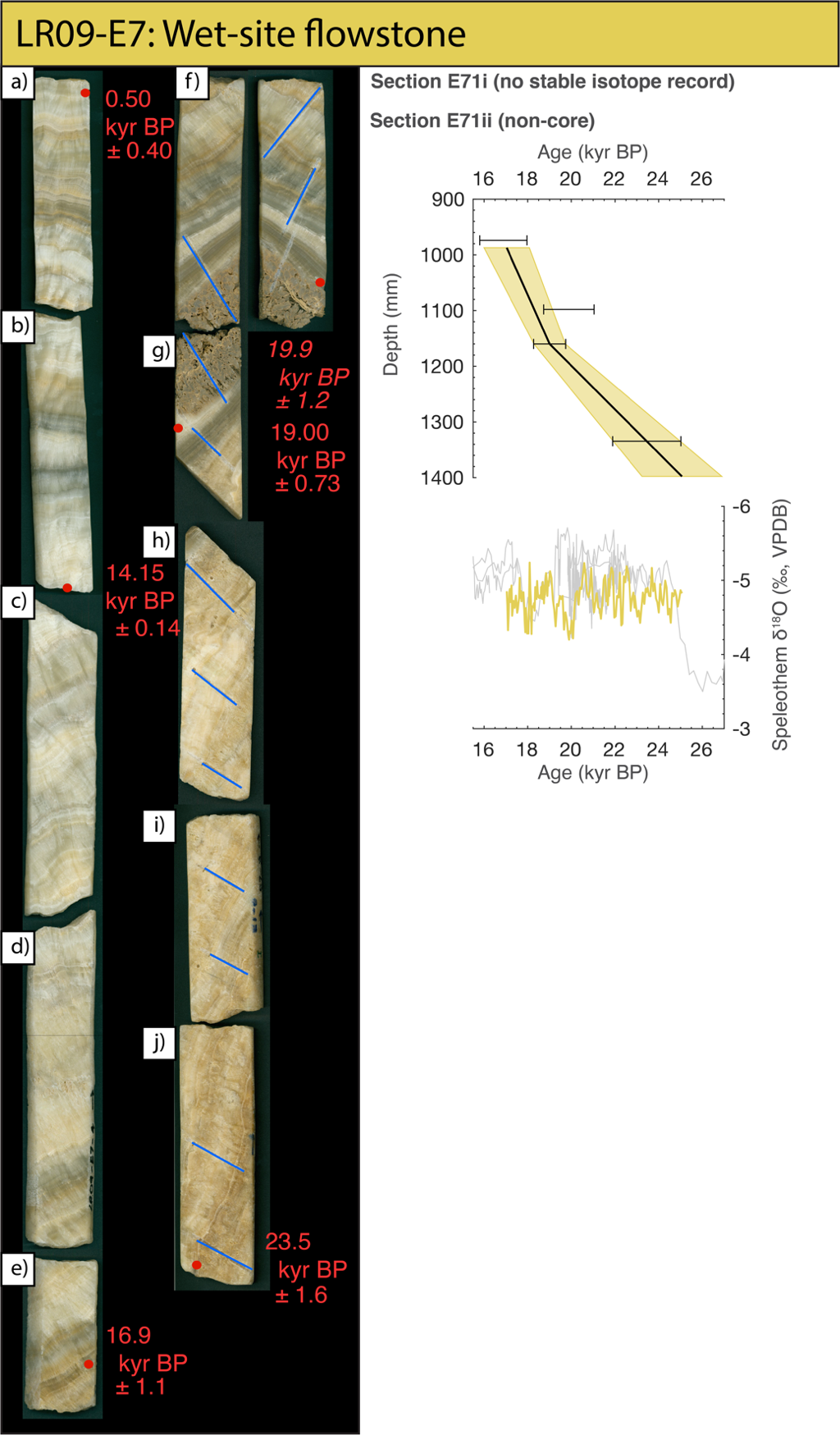


**Figure S8:** LR09-E7 is a flowstone that grew from ~25 kyr BP to the present in what is thought to be the wettest part of the cave. It was drilled to recover drier periods before and after the Last Glacial Maximum, which are typically recorded by hiatuses or condensed aragonite sections in the cave’s stalagmites. LR09-E7 was initially drilled to a depth of 986 mm in 2009 in five sections covering 17 to 0 kyr BP. A further five sections were recovered in 2011, to a depth of 1399 mm, extending the record to ~25 kyr BP. It was these deeper five sections that were analysed for stable isotopes. The quality of calcite in the top half of the flowstone is good, with fine laminae common. A section of poor-quality calcite is bracketed by two reversed dates of 19.9 and 19.0 kyr BP. Below this section the quality of calcite is poorer and more opaque, but it retains the fine laminae throughout.

**Supplementary Discussion: Age model and composite record construction**

Individual age models for all stalagmites were constructed using ISCAM (Fohlmeister, 2012). First, for each individual speleothem, 100,000 Monte Carlo simulations produced linearly interpolated age models using random ages drawn from a Gaussian distribution defined by the U-Th dates. Each section of stalagmite (i.e., between hiatuses) was treated as a separate entity, with a total of 32 separate sections from 15 individual speleothems.

Of these 32 sections, 13 sections from eight, high quality, long growth period stalagmites were chosen to produce the composite record. They are:

LR06-B1i, LR06-B1ii LR06-B1iii (sections 1-3 of 3)

LR06-B3i (section 1 of 2)

LR06-C2i (section 1 of 1)

LR06-C5i LR06-C5ii (sections 1-2 of 2)

LR09-N1i (section 1 of 2)

LR06-C6iii LR06-C6iv (sections 3-4 of 4)

LR11-K5ii LR11-K5iii (sections 2-3 of 3)

LR09-J1i (section1 of 1)

ISCAM produces an optimised age model for each speleothem record through a correlation procedure, whereby the linear age models are allowed to vary between 2σ uncertainty to achieve the best correlation across intervals of overlap. First, individual age models were smoothed to 10-year resolution to prevent fast growth-rate speleothems with high-frequency variation in δ^18^O dominating the record. Correlation proceeds sequentially back through time, correlating older speleothem #2 to speleothem #1, and then the next oldest speleothem #3 to the composite of #1 and #2.

We used ISCAM settings of 100,000 Monte Carlo simulations, and 200 first-order autoregressive (AR1) simulations with 100 Monte Carlo simulations per AR1. The AR1 process is used for error estimation using red noise simulated records. We do not report the ISCAM estimated error as we believe it is too constrained relative to the age uncertainty of the individual records, and therefore the number of AR1 simulations (200) is relatively low. Additional ISCAM settings include a 10-year smoothing, assumed Gaussian error on the U-Th ages, pointwise linear interpolation for the initial age models, no detrending of individual records, and allowing extrapolation of age models beyond ages to the top/bottom of stalagmite sections (typically only a few mm, as our U-Th sampling strategy focused on hiatus detection by targeting changes in speleothem texture).

As ISCAM progresses sequentially, composites can be subject to cumulative age errors when many speleothem sections are added. Frequently the age model is forced to the extremes of the 2σ linearly interpolated error to provide the best correlation, and therefore repeating this process numerous times can result in the composite no longer fitting individual speleothem ages accurately. This also results in arbitrarily small uncertainty on the timing of the composite as all simulations are forced similarly. In our case, limiting the number of speleothem sections used in the composite to only the 13 highest quality, replicating speleothem sections reduces the cumulative error; compositing 30 speleothem sections would likely introduce significant error.

ISCAM struggles to correlate speleothem sections with a low number of U-Th age determinations (three or less), as one of two scenarios typically occur. If the ages overlap, ISCAM determines that the fastest possible growth rate is the most likely, as statistically, higher correlations occur with shorter overlapping sections. Alternatively, age models are pulled to one extreme value of the 2σ uncertainty envelopes. This is because a better/the best correlating age model may lie outside the 2σ uncertainty envelope, and therefore ISCAM pulls the age model as far as it is allowed. This is an inherent disadvantage in searching for an optimal statistical measure in a Monte-Carlo simulation, rather than a ‘most likely scenario’ used by most other age model software packages. These scenarios tend not to occur when there are four or more U-Th age determinations (69% of the core stalagmite age models remain within the 1σ uncertainty envelope of U-Th age determinations at the relevant depths) . Therefore, we caution against the use of ISCAM as age modelling software on speleothem sections with only 2 or 3 U-Th age determinations.

Accordingly, we used the ISCAM-derived linearly interpolated age model for the 14 (out of 19) non-core speleothem sections with multiple age determinations and stable isotope transects. We did calculate ISCAM-adjusted age models for these sections, running each section individually as speleothem #2 with the composite as speleothem #1. However, we found the resulting age models to be untrustworthy. The 14 non-core speleothem sections are:

LR06-B3ii (section 2 of 2)

LR06-C3i LR06-C3ii (sections 1-2 of 2)

LR11-C8ii (section 2 of 2)

LR07-E1i LR07-E1ii (sections 1-2 of 2)

LR09-E7ii (section 2 of 2)

LR09-G4i LR09-G4ii LR09-G4iii (sections 1-3 of 4)

LR11-G7i LR11-G7ii (sections 1-2 of 2)

LR09-L1i LR09-Lii (sections 1-2 of 2)

No age model was calculated for the final five speleothem growth sections because they have either no stable isotope data if their time-periods are well covered by other material (e.g. LR09-E7 – section 1, LR11-C8 – section 1), have overlapping ages that don’t allow ISCAM to produce an age model greater than 50 years (e.g. LR09-N1 section 2), or have only one U-Th date, preventing accurate age model construction (LR11-K5 – section 1, LR09-G4 – section 4).

ISCAM also has the option to adjust speleothem δ^18^O (i.e., in the ‘y-direction’) to achieve overlap using a non-constant warping across overlapping periods. Again, using many speleothem sections introduces cumulative errors such that δ^18^O values at the start of the record are not comparable to δ^18^O at the end of the record. We therefore did not use this feature of ISCAM.

A final, evenly spaced, composite record with 50-year resolution was produced in MATLAB. Briefly, δ^18^O values for the core records were interpolated to 1-year resolution using a piecewise cubic Hermite interpolating spline. This creates a weighted average throughout each 50-year bin that ensures that anomalous points are not over- or under-represented; areas of low resolution have full data coverage while areas of high-resolution are not over-represented in the composite record. The 1-year interpolated record is then averaged to the mid-point of each 50-year bin to create a universal timescale. The start and end δ^18^O values for of each record was screened to avoid overly large excursions caused by extrapolation by the spline, or by kinetic fractionation on a drying drip (i.e., large positive isotopic trends). Finally, all the individual records are averaged across their intervals of overlap.

Overall, the composite record provides a good balance between adhering to isotopic changes in stalagmite records and producing an averaged record when there is conflicting information for intervals of overlap between coeval stalagmites. However, we note that the composite may be weak around 20 kyr BP. At this point the δ^18^O values for LR06-C5 and LR06-C6 are offset by ~0.5‰ for a few hundred years.

To avoid the introduction of any bias or smoothing to the record that the regularly spaced composite might produce, discussion of millennial scale variability in the text focuses on individual records (with the original δ^18^O values) rather than the composite.


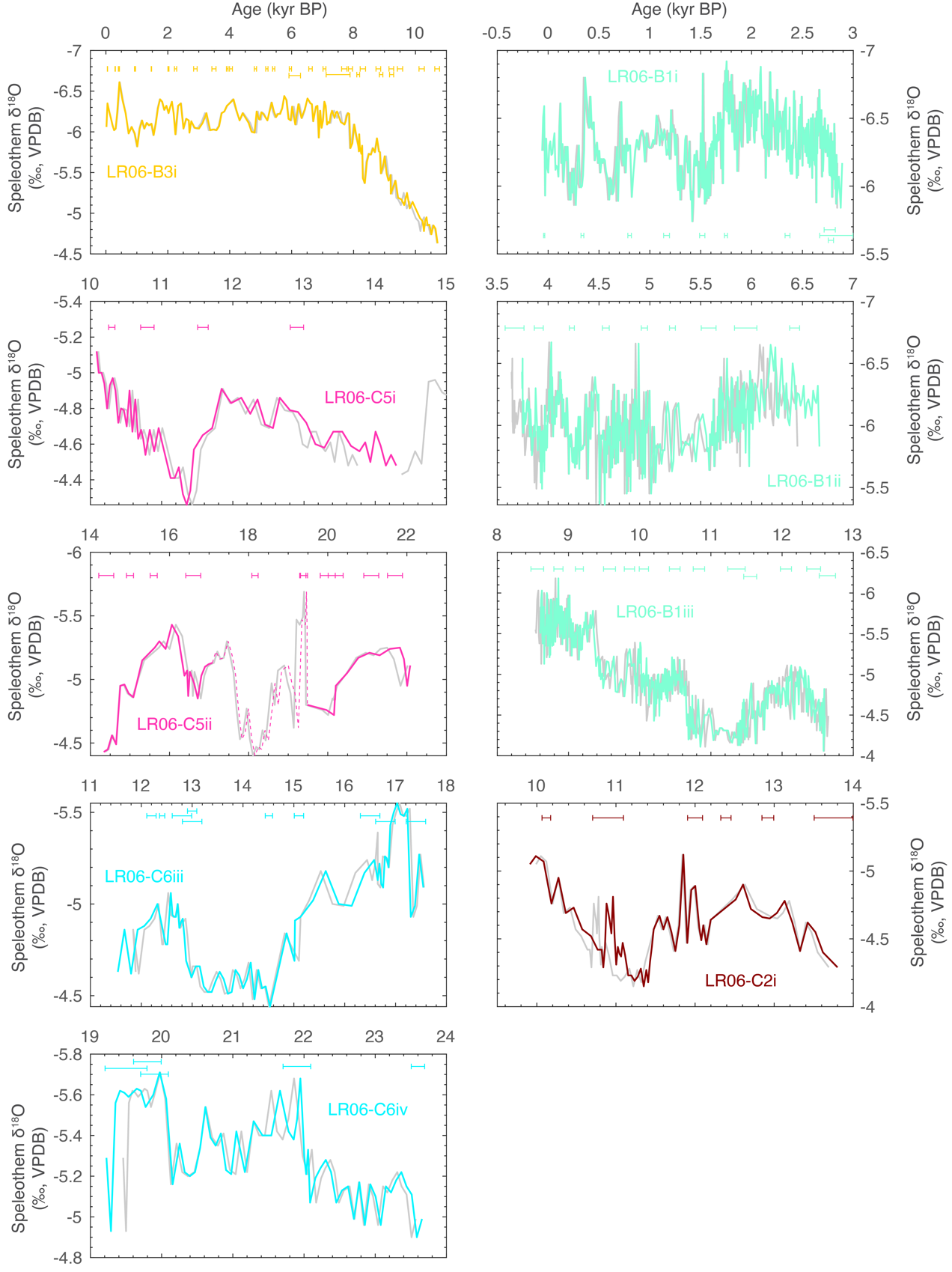


**Figure S9:** ISCAM models for core sections of stalagmites previously published in Griffiths et al., (2009) and Ayliffe et al., (2013). Bars indicate U-Th ages with 2σ uncertainties. Grey lines indicate age model in original publication. Coloured lines show new ISCAM age models. In general, there is little difference between the previously published and updated age models. The gap between LR06-C5i and LR06-C5ii is considerably smaller than that identified by Ayliffe et al., (2013), and could be interpreted as a growth-rate slowdown by age-modelling software. However, as Ayliffe et al., (2013) identified an unconformity at this ‘gap’, we stick to the original interpretation of there being a small hiatus in this record. Dashed line in LR06-C5ii indicates presence of both calcite and aragonite mineralogies.

**
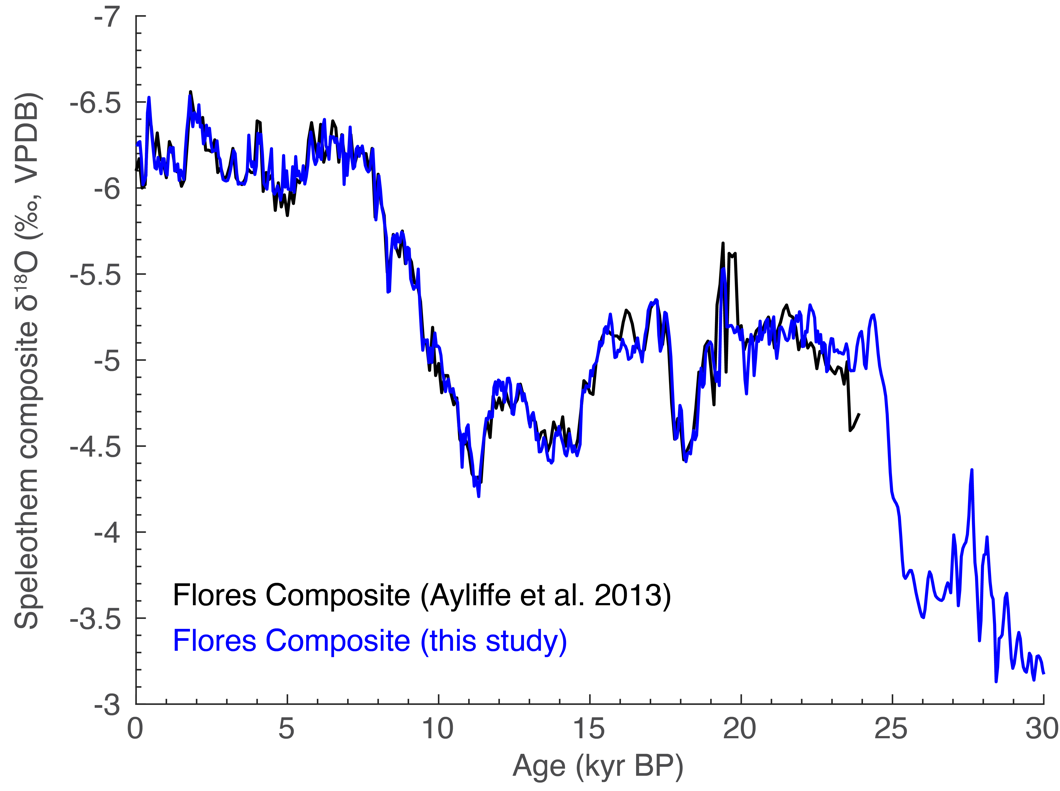
**

**Figure S10:** Comparison of the Ayliffe et al., (2013) composite with the new composite. The previous composite (linear age models spliced together at 100-year resolution) and the new extended composite (ISCAM age models, interpolated, binned, and averaged at 50-year resolution) are in good agreement. The only significant area of disagreement is between 20 and 19.6 kyr. The ISCAM age model has closed a small unconformity in LR06-C5 (Ayliffe et al. 2013) across this 400-year interval. In the new composite, LR06-C5 is shifted younger to fill the gap and is averaged with LR06-C6.


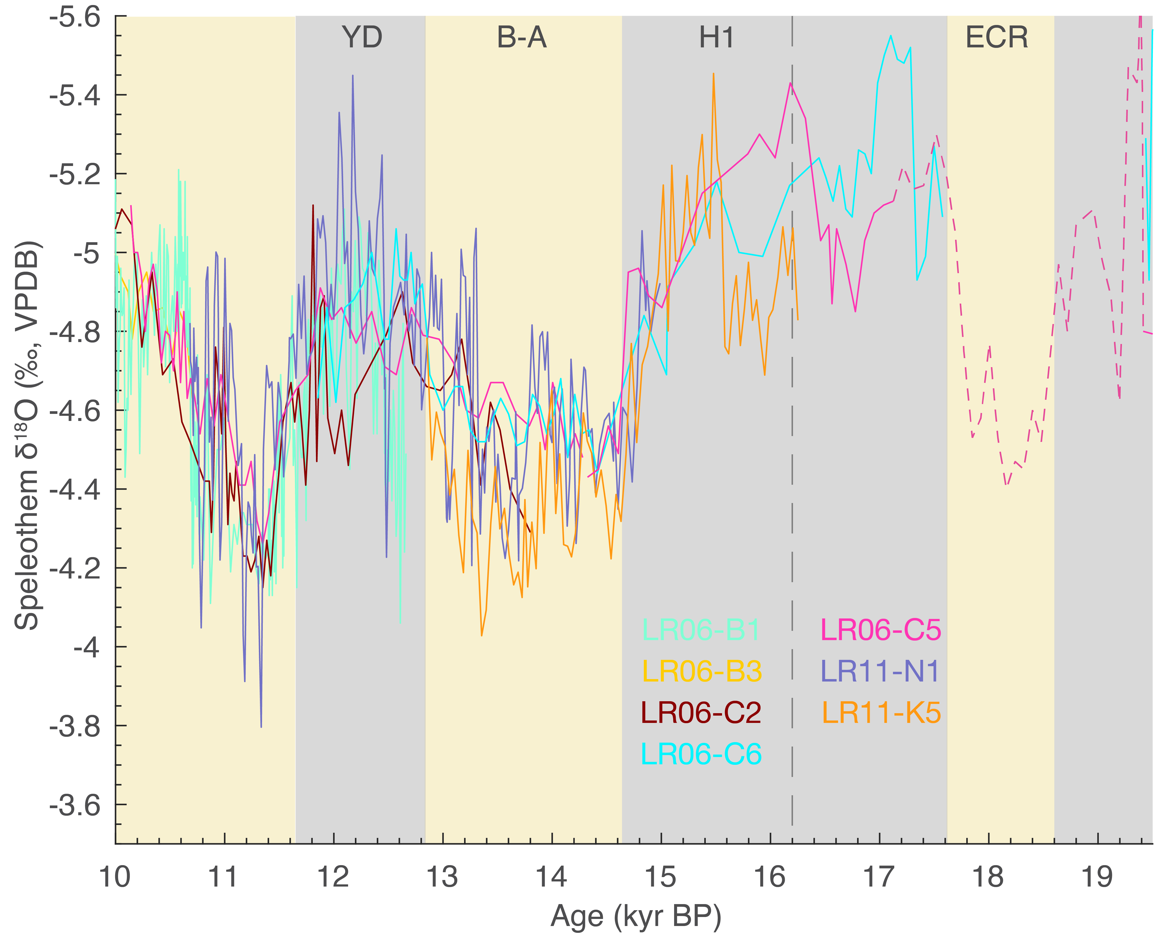


**Figure S11:** Replication of core stalagmite δ^18^O records during the deglaciation. The records for 19.5 and 10 kyr BP show excellent agreement through the millennial scale events of the deglaciation. YD = Younger Dryas, B-A = Bølling-Allerød, H1 = Heinrich event 1, ECR = Extrapolar Climate Reversal. The δ^18^O values for the calcite-aragonite section of LR06-C5 (dashed line) have been isotopically to calcite-equivalent values using a calcite/aragonite mass balance (Ayliffe et al., 2013).


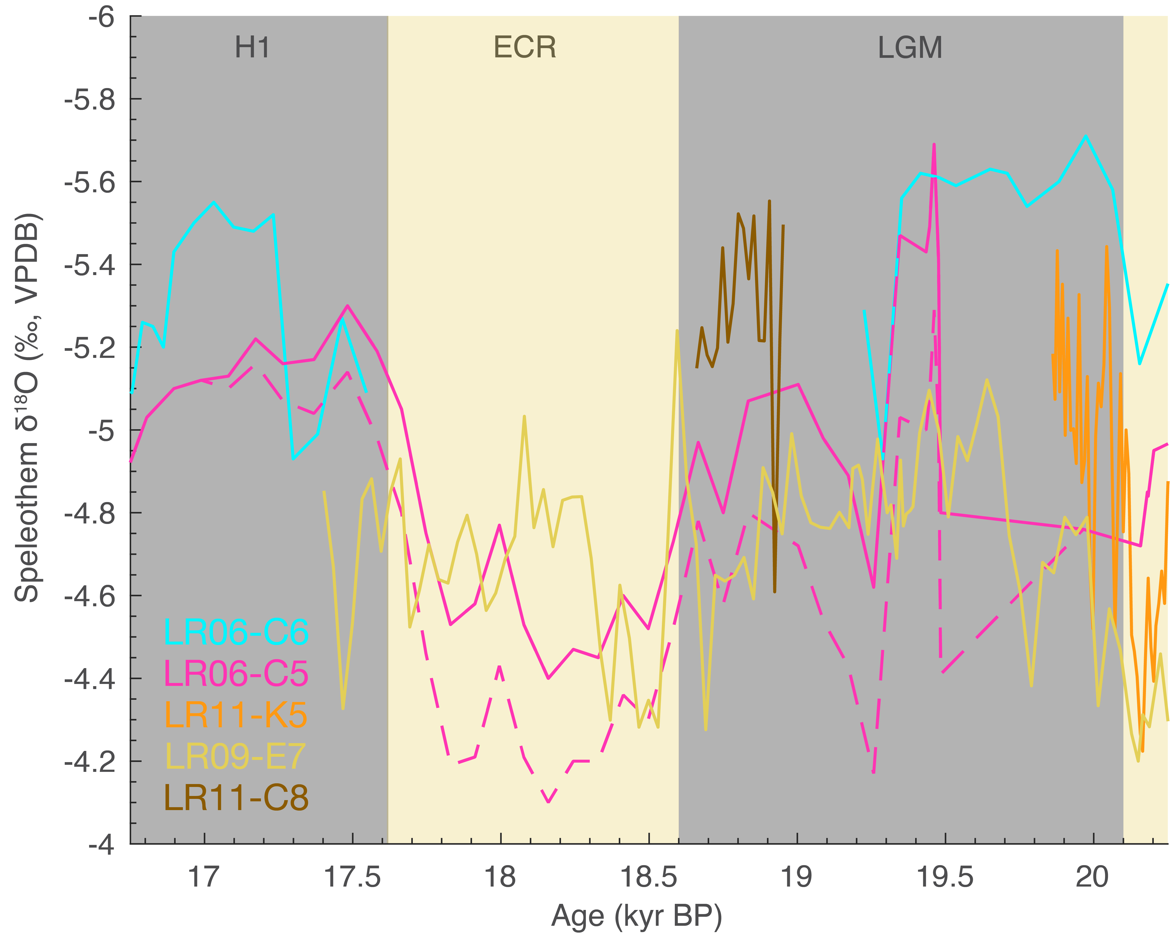


**Figure S12:** Replication of core and non-core speleothem δ^18^O records during the Extrapolar Climate Reversal. LR06-C5 is composed of a mixture of calcite and aragonite between 19.5 and 17.1 kyr BP (dashed pink line). Ayliffe et al., (2013) applied a proportional adjustment to the δ^18^O values based on the amount of aragonite and calcite detected by 10 x-ray diffraction measurements across this interval (solid pink line). This section contains the Extrapolar Climate Reversal. None of the new core speleothem overlap this interval, but non-core speleothem sections do. LR09-E7 is a flowstone core (Fig. S8) with uncertain chronology around this time, but the δ^18^O values around -4.6 to -4.8 ‰ suggest that the LR06-C5 correction is appropriate. LR11-C8 (Fig. S5) provides further evidence for lower δ^18^O during the LGM, as does the top of LR11-K5. These results lend further weight to the evidence for the ECR being relatively dry in Flores, and the LGM being relatively wet.

**References:**

Ayliffe, L.K., Gagan, M.K., Zhao, J.-x., Drysdale, R.N., Hellstrom, J.C., Griffiths, M.L., Pierre, E.S., Hantoro, W.S., Cowley, J., Scott-Gagan, H., and Suwargadi, B.W. Rapid interhemispheric climate links via the Australasian monsoon during the last deglaciation. *Nat. Commun.*, 42908, <https://doi.org/10.1038/ncomms3908> (2013).

Fohlmeister, J. A statistical approach to construct composite climate records of dated archives. *Quat. Geochronol.*, https://doi.org/1448–56, 10.1016/j.quageo.2012.06.007 (2012).

Griffiths, M.L. *et al*. Increasing Australian-Indonesian monsoon rainfall linked to early Holocene sea-level rise. *Nat. Geosci.*, 2(9), 636–639, https://doi.org/10.1038/ngeo605 (2009).

Tarutani, T., Clayton, R.N., and Mayeda, T.K. The effect of polymorphism and magnesium substitution on oxygen isotope fractionation between calcium carbonate and water. *Geochim. Cosmochim. Acta.*, 33(8), 987–996, https://doi.org/10.1016/0016-7037(69)90108-2 (1969).

Kim, S.-T., O’Neil, J.R., Hillaire-Marcel, C., Mucci, A. Oxygen isotope fractionation between synthetic aragonite and water: Influence of temperature and Mg2+ concentration. *Geochim. Cosmochim. Acta.*, 71, 4704–4715, https://doi.org/10.1016/j.gca.2007.04.019 (2007)
